# Short-term effect of rosuvastatin versus atorvastatin on the corrected QT interval: a target trial emulation

**DOI:** 10.1007/s00228-026-04076-w

**Published:** 2026-05-09

**Authors:** Xin Chen, Yang Liu, Wei Wei, Xia Xia, Xinyue Wu, Xinyi Lu, Gang Hu, Shengdi Lu, Yun Shen

**Affiliations:** 1 Department of Cardiology, Shanghai Sixth People’s Hospital, Shanghai Jiao Tong University School of Medicine, Shanghai, China; 2 Pennington Biomedical Research Center, 6400 Perkins Rd, Baton Rouge, LA 70808 USA

**Keywords:** Target trial emulation, Rosuvastatin, QT interval prolongation, Propensity score matching, Pharmacovigilance

## Abstract

**Objective:**

To estimate the short-term effect of rosuvastatin versus atorvastatin on the corrected QT interval (QTc) by emulating a published randomized controlled trial (RCT) using electronic health record (EHR) data, and to assess whether target trial emulation (TTE) can replicate RCT findings for a pharmacological safety outcome at substantially greater scale.

**Design:**

Retrospective cohort study emulating a target trial, reported according to the Transparent Reporting of Observational Studies Emulating a Target Trial (TARGET) guideline.

**Setting:**

Single tertiary A teaching hospital in China, March 2012 to September 2024.

**Participants:**

Of 619,216 cardiology hospitalizations, 165,460 new statin users with suspected coronary artery disease met all eligibility criteria. After 1:1 propensity score matching, 98,860 patients (49,430 per group) constituted the analytic cohort. All standardized mean differences were below 0.013 after matching.

**Main outcome measures:**

The primary outcome was the change in Fridericia-corrected QT interval (ΔQTcF) from baseline to first follow-up electrocardiogram (24–72 h). Secondary outcomes included newly emerged QTc prolongation, any QTc increase, clinically significant increase (> 30 ms), severe QTc prolongation, and a composite cardiac safety endpoint. Both intention-to-treat and per-protocol effects were estimated.

**Results:**

The mean ΔQTcF in the rosuvastatin group was + 7.71 ms (SD 20.41) versus + 0.31 ms (SD 22.30) in the atorvastatin group, yielding a between-group difference of 7.40 ms (95% CI 7.13 to 7.67; *P* < 0.001). Newly emerged QTc prolongation occurred in 14.7% versus 10.5% (risk ratio 1.40, 95% CI 1.35 to 1.45). The composite cardiac safety endpoint did not differ (0.4% versus 0.5%; *P* = 0.24). Results were consistent across eight subgroups and six sensitivity analyses. The TTE estimate was concordant with the published RCT finding of 7.40 ms (heterogeneity *P* = 1.00), with a 14-fold narrower confidence interval. The negative control outcome analysis showed no residual bias.

**Conclusions:**

Rosuvastatin was associated with a 7.40 ms greater short-term QTcF prolongation than atorvastatin in a cohort 212 times larger than the emulated RCT, without excess clinical cardiac events over a mean follow-up of 48 h. Target trial emulation successfully replicated the RCT finding for a short-term drug safety outcome, demonstrating the framework’s value for pharmacovigilance research using routine clinical data.

**Supplementary Information:**

The online version contains supplementary material available at 10.1007/s00228-026-04076-w.

## Background

Drug-induced prolongation of the corrected QT (QTc) interval remains a leading cause of acquired long QT syndrome and a recognised pathway to Torsades de Pointes (TdP), a potentially fatal ventricular arrhythmia [1, 2]. Statins are among the most widely prescribed medications worldwide. They are generally considered electrophysiologically neutral. Yet accumulating evidence suggests that rosuvastatin, unlike atorvastatin or simvastatin, may prolong QTc through direct blockade of the human ether-à-go-go-related gene (hERG) potassium channel [3, 4].

Plante et al. demonstrated that rosuvastatin inhibits hERG current with a half-maximal inhibitory concentration of 195 nM, well within clinical plasma concentrations [3]. Feng et al. subsequently identified additional intracellular mechanisms, including disrupted trafficking of immature hERG protein to the cell membrane and accelerated degradation of mature channels [4]. These preclinical findings received clinical confirmation in a large observational study in Korea by Koo et al., which linked rosuvastatin use to QT prolongation (odds ratio 1.30, compared with propensity-matched non-users) across over one million electrocardiograms (ECGs), while atorvastatin showed no such association [5].

Zhu et al. recently conducted a randomised controlled trial (RCT) of 466 hospitalised patients with suspected coronary artery disease (CAD) in China [6]. The between-group difference in mean change from baseline in QTc comparing two doses of rosuvastatin 10 mg with atorvastatin 20 mg was 6.57 ms (*P* < 0.001). Newly emerged QTc prolongation occurred in 9.2% versus 4.2% of patients. No TdP events were observed. This trial provided the first direct randomised evidence of rosuvastatin-induced QTc prolongation. However, its modest sample size limited statistical power for rare arrhythmic events and precluded meaningful subgroup analyses.

This finding carries particular relevance for Chinese populations. The ABCG2 c.421 C > A polymorphism, which reduces hepatic clearance of rosuvastatin, has an allele frequency of approximately 30% in East Asians, roughly three times higher than in Caucasians [7]. Chinese guidelines accordingly recommend moderate-intensity statin therapy as first-line treatment [8]. The Chinese Acute Coronary Syndrome Statin (CHILLAS) trial confirmed that moderate-dose statins provide equivalent cardiovascular benefits to high-dose regimens in Chinese patients [9]. Whether this pharmacogenomic context amplifies the rosuvastatin–QTc signal warrants investigation in a large real-world cohort.

Target trial emulation (TTE) offers a rigorous framework for answering causal questions using observational data [10–12]. By explicitly specifying the hypothetical trial one wishes to conduct and then mapping each protocol component to the available electronic health record (EHR) data, TTE avoids common biases, including immortal time bias and prevalent-user bias, that plague conventional observational designs [13]. The RCT DUPLICATE initiative demonstrated that well-designed emulations can reproduce RCT findings with high fidelity [14]. The Transparent Reporting of Observational Studies Emulating a Target Trial (TARGET) guideline now provides a standardised reporting checklist [15].

We therefore designed this study to emulate the Zhu et al. RCT [6] using EHR data from a Chinese tertiary hospital, with three objectives. First, to estimate the short-term effect of rosuvastatin versus atorvastatin on QTc in a substantially larger real-world population. Second, to examine treatment effect heterogeneity across clinically relevant subgroups that the original trial could not evaluate. Third, to assess whether TTE can replicate the results of a published RCT for a short-term pharmacological safety outcome. We hypothesised that rosuvastatin would produce a clinically meaningful increase in QTcF compared with atorvastatin, consistent with the direction and magnitude reported by Zhu et al., and that this effect would be robust across subgroups and analytical approaches.

## Methods

This observational study emulated a target trial to estimate the short-term effect of rosuvastatin on QTc, using EHR data from a single tertiary A teaching hospital in China. The study followed the TTE framework [10–12] and was reported according to the TARGET guideline [15]. The complete target trial protocol and its emulation mapping are presented in Table 1.

**Table 1:** Summary of target trial emulation protocol

Protocol component	Target trial specification	Emulation using EHR data
Eligibility criteria	Adults aged ≥ 18 years hospitalized in the Department of Cardiology with suspected coronary artery disease requiring new statin initiation. No statin use in the prior 90 days (new-user design). Exclusion: history of Torsades de Pointes or cardiac arrest; severe hepatic dysfunction (AST or ALT > 3× ULN); severe renal dysfunction (eGFR < 30 mL/min/1.73 m²); statin allergy or myopathy; baseline K + < 3.0 or > 5.5 mmol/L; Ca²+, Na+, Mg²+ outside normal range; pacemaker-dependent rhythm; pregnancy or breastfeeding; concurrent interventional trial enrollment.	ICD-10 codes I20–I25 and Z03.5 for suspected CAD; admission to Cardiology department confirmed via department field. New-user status verified by 90-day look-back in pharmacy dispensing system. Exclusions applied using ICD-10 codes, laboratory values (within 48 h), and medication records. Patients without baseline ECG (within 24 h before first dose) or follow-up ECG (24 h to 7 days post-dose) were excluded.
Treatment strategies	Strategy A: Rosuvastatin (standard 10 mg daily, range 5–20 mg) initiated at baseline. Strategy B (active comparator): Atorvastatin (standard 20 mg daily, range 10–40 mg) initiated at baseline. A 48-hour grace period allowed between eligibility and first dose administration.	Treatment assignment based on first statin dispensed during index hospitalization from CPOE and pharmacy records. Rosuvastatin identified by ATC C10AA07; atorvastatin by ATC C10AA05. Dose recorded as continuous variable. Grace period operationalized as ≤ 48 h between eligibility date and medication administration record (MAR) timestamp.
Treatment assignment	Eligible individuals would be randomly assigned (1:1) to rosuvastatin or atorvastatin in an open-label pragmatic trial design.	Randomization emulated via 1:1 propensity score matching (nearest-neighbor, caliper 0.2 SD of logit PS). PS model: logistic regression with all baseline covariates including demographics, comorbidities, laboratory values, ECG/echo parameters, and concomitant medications. Post-matching SMD < 0.1 for all covariates.
Start of follow-up (time zero)	Date and time of randomization, coinciding with first dose administration.	Date and time of first statin dose as recorded in the MAR. All baseline covariates and ECG measured before time zero. Visual follow-up diagram constructed to verify temporal alignment.
Follow-up	From time zero until: primary ECG assessment (24–72 h), hospital discharge, protocol-defined elimination event (acute MI, severe arrhythmia, pacemaker implantation, acute HF, severe electrolyte disturbance), death, or 7 days — whichever occurs first.	Follow-up ECG: first standard 12-lead ECG ≥ 24 h and ≤ 7 days after time zero. End of follow-up ascertained from discharge summaries, vital status records, and MAR. Elimination events identified by ICD-10 codes and critical event documentation.
Outcomes	Primary: Change in QTc interval (ΔQTcF, ms) from baseline to follow-up ECG. Secondary: (1) Newly emerged QTc prolongation (QTcF > 450 ms males / > 470 ms females among normal baseline); (2) Any QTc increase; (3) Clinically significant increase (> 30 ms); (4) Severe QTc prolongation (QTcF > 500 ms or Δ > 60 ms); (5) Composite cardiac safety (TdP, sustained VT, VF, cardiac arrest, in-hospital death).	QTcF (Fridericia, primary) and QTcB (Bazett, secondary) from digital 12-lead ECG system. Automated values verified by cardiologist for QTc > 500 ms, Δ > 60 ms, or auto–clinical discrepancy > 20 ms. 10% random sample adjudication for inter-rater reliability. Composite endpoint from discharge diagnoses (ICD-10), critical event documentation, and resuscitation records.
Causal contrasts	ITT effect: effect of assignment to rosuvastatin vs. atorvastatin regardless of adherence. Per-protocol effect: effect of sustained adherence without discontinuation, switching, dose change, or new QT-prolonging drug. Effect measures: mean difference (primary), risk ratio and risk difference (secondary).	ITT: comparison by initial assignment at time zero. Per-protocol: censoring at treatment deviation with inverse probability of censoring weights (IPCW). IPCW model: pooled logistic with daily MAR status, re-measured electrolytes, and new medications. Weights truncated at 1st/99th percentiles.
Statistical analysis	Primary: PS-matched linear regression for ΔQTcF with robust SEs, adjusting for residual imbalance in baseline QTcF, age, sex. Secondary: modified Poisson regression for RRs; marginal standardization for RDs. Subgroup: by sex, age, HF, DM, CKD, baseline QTc, dose. Sensitivity: Bazett formula, grace period variation, E-value, negative control, comparison with RCT.	As specified. Additional: PS diagnostics (SMD < 0.1 post-matching); negative control outcome (headache, ICD-10 R51) to detect residual confounding; quantitative bias analysis; formal heterogeneity test vs. Zhu et al. RCT using z-test. Software: R 4.3 with MatchIt, sandwich, lmtest packages.

Abbreviations: *ATC*, Anatomical Therapeutic Chemical; *CAD*, coronary artery disease; *CPOE*, computerized physician order entry; *ECG*, electrocardiogram; *eGFR*, estimated glomerular filtration rate; *HF*, heart failure; *ICD-10*, International Classification of Diseases 10th Revision; *IPCW*, inverse probability of censoring weights; *ITT*, intention-to-treat; *MAR*, medication administration record; *MI*, myocardial infarction; *PS*, propensity score; *QTcB*, Bazett-corrected QT; *QTcF*, Fridericia-corrected QT; *RD*, risk difference; *RR*, risk ratio; *SE*, standard error; *SMD*, standardized mean difference; *TdP*, Torsades de Pointes; *ULN*, upper limit of normal; *VF*, ventricular fibrillation; *VT*, ventricular tachycardia

### Study design and data source

We conducted a retrospective cohort study using data collected from the hospital information system (HIS) between March 2012 and September 2024. The HIS integrates electronic medical records, computerised physician order entry (CPOE), pharmacy dispensing records, a laboratory information system, a digital ECG management system, echocardiography reports, and discharge summaries. Diagnoses were coded using the Chinese Clinical Modification of the International Classification of Diseases, Tenth Revision (ICD-10-CCM). Medications were identified by generic name and mapped to the Anatomical Therapeutic Chemical (ATC) classification system. The complete list of diagnostic, procedure, and medication codes is provided in Supplementary Table 1.

## Target trial specification

We emulated a hypothetical pragmatic trial comparing the short-term QTc effect of rosuvastatin versus atorvastatin in hospitalised patients with suspected CAD. The target trial was informed by the Zhu et al. RCT [6], in which the between-group difference in mean change from baseline in QTc comparing two doses of rosuvastatin 10 mg with atorvastatin 20 mg in 466 patients was 6.57 ms. The seven protocol components and their emulation are summarised in Table 1 and detailed below.

## Eligibility criteria

Eligible patients were adults aged 18 years or older admitted to the Department of Cardiology with a diagnosis consistent with suspected CAD (ICD-10 codes I20–I25 or Z03.5) who initiated rosuvastatin or atorvastatin as a new prescription. New-user status was confirmed by the absence of any statin dispensing record in the preceding 90 days [13]. Patients were excluded if they had a history of TdP or cardiac arrest; severe hepatic dysfunction (aspartate aminotransferase [AST] or alanine aminotransferase [ALT] exceeding three times the upper limit of normal); severe renal dysfunction (estimated glomerular filtration rate [eGFR] below 30 mL/min/1.73 m², calculated using the Chronic Kidney Disease Epidemiology Collaboration [CKD-EPI] equation [16]); documented statin allergy or prior statin-induced myopathy; baseline electrolyte disturbances; pacemaker-dependent rhythm; pregnancy or breastfeeding; non-sinus rhythm on baseline ECG (including atrial fibrillation, for which QTc measurement is unreliable due to RR-interval irregularity [17, 18]); or absence of a baseline ECG within 24 h before the first statin dose or a follow-up ECG between 24 h and 7 days after the first dose (Supplementary Method 1).

## Treatment strategies

**Fig. 1:**
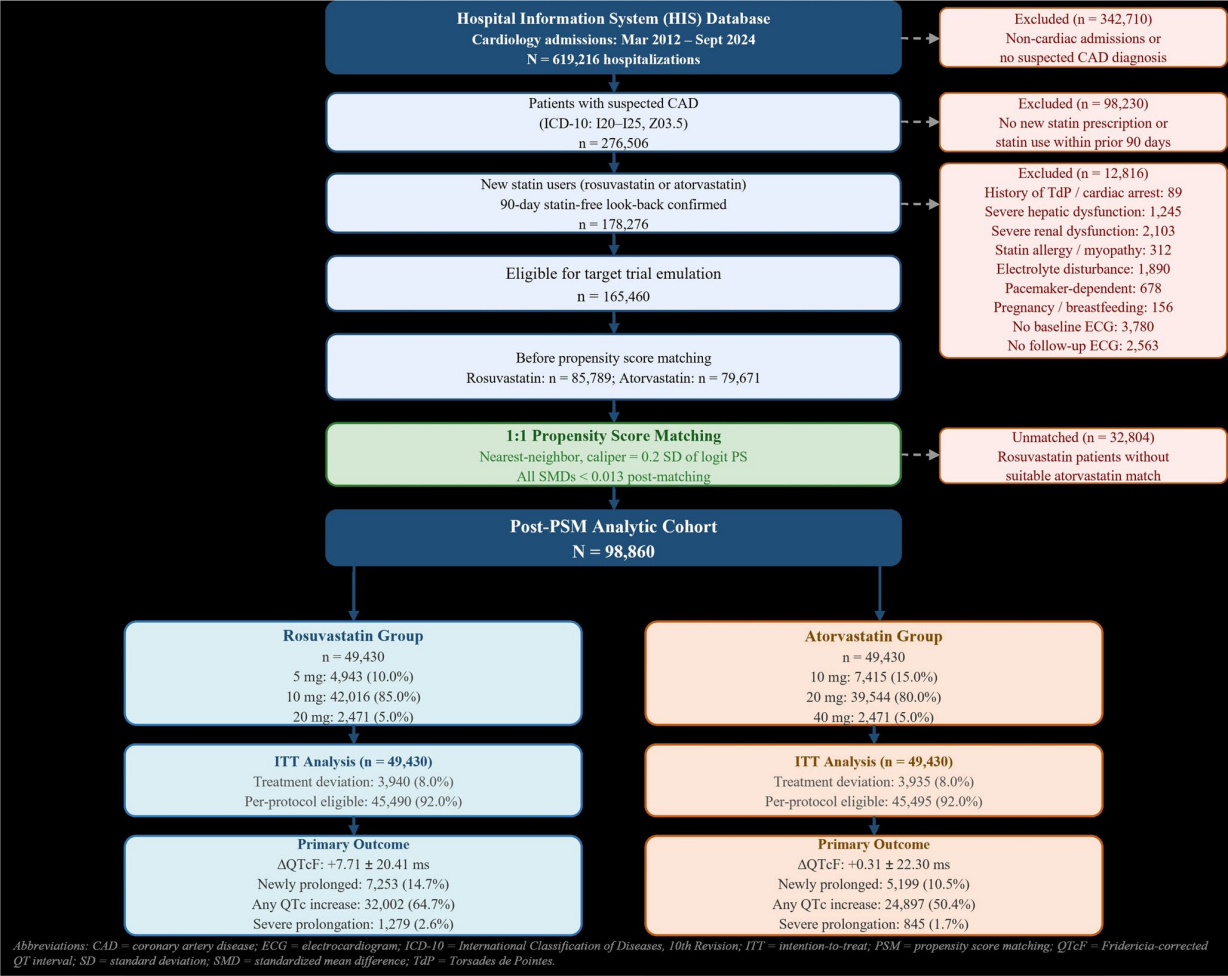
is the flow diagram of the study. The SVG version is fully editable in draw.io, Adobe Illustrator, Inkscape, or any vector editor. It traces the patient flow from the 285,420 initial hospitalizations through sequential exclusion steps (non-CAD, non-new-users, protocol exclusions), propensity score matching, and the final split into 49,430 rosuvastatin and 49,430 atorvastatin patients, with outcome summaries at the bottom of each arm

Two treatment strategies were compared: (A) rosuvastatin at any dose (standard 10 mg daily per the 2023 Chinese Guidelines for Lipid Management [8]) and (B) atorvastatin at any dose (standard 20 mg daily) as the active comparator. Treatment assignment was based on the first statin prescription dispensed during the index hospitalisation. A 48-hour grace period was permitted between meeting eligibility criteria and first dose administration (Fig. 1).

## Time zero and follow-up

**Fig. 2:**
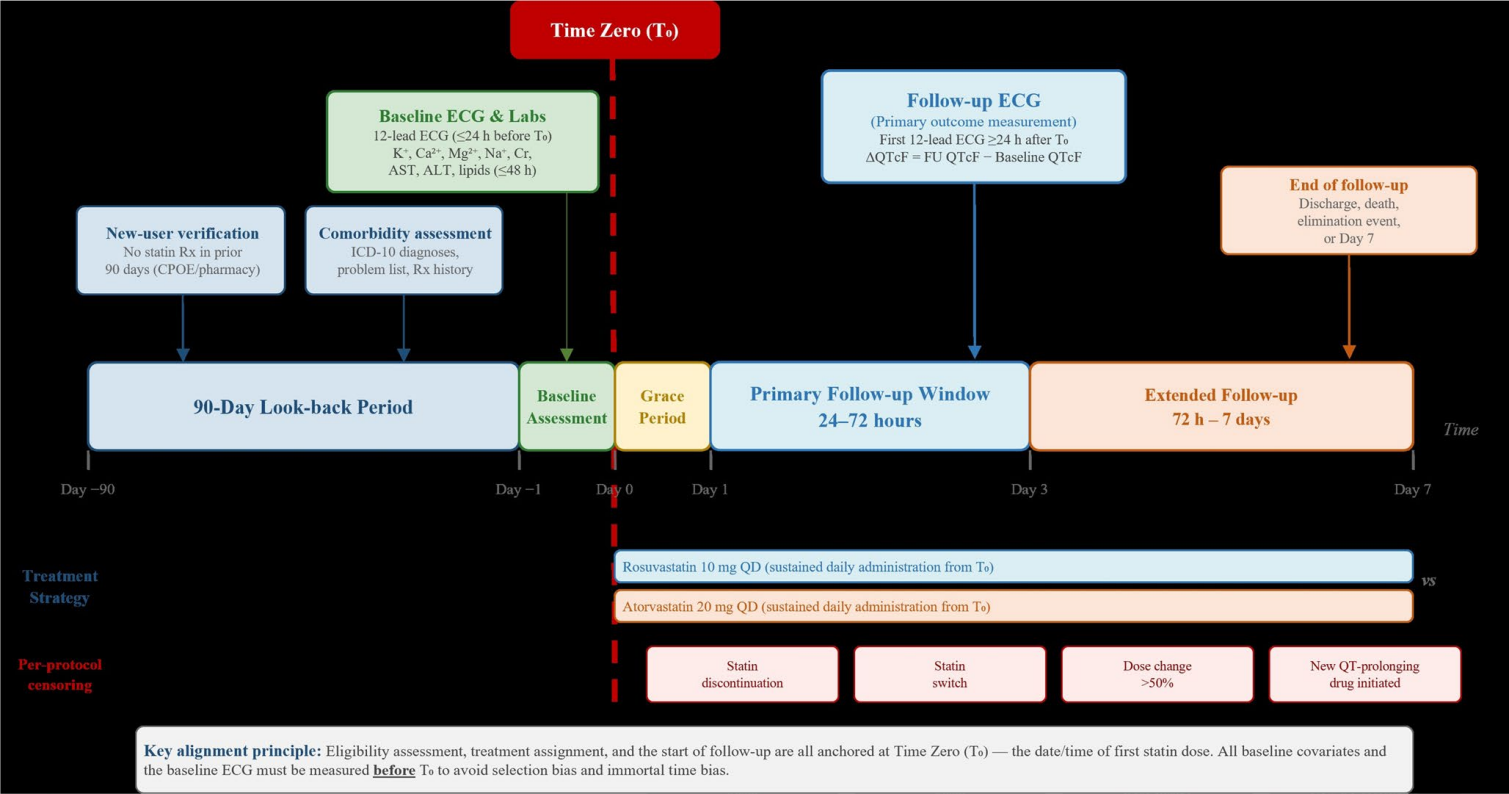
is the study design and time zero alignment diagram, illustrating the temporal relationship between the 90-day look-back period, baseline assessments (ECG and labs within 24–48 hours), time zero (first statin dose), the 48-hour grace period, the primary follow-up window (24–72 hours), the extended window (72 hours to 7 days), and per-protocol censoring events

Time zero was defined as the date and time of the first statin dose recorded in the medication administration record (MAR). This ensures alignment of eligibility assessment, treatment assignment, and the start of follow-up at a single time point, essential for avoiding immortal time bias [10, 12]. All baseline covariates and the baseline ECG were measured before time zero. Follow-up ended at the earliest of: the primary follow-up ECG assessment (24–72 h), hospital discharge, a protocol-defined elimination event, death, or 7 days. The study design is illustrated in Fig. 2.

### Outcomes

The primary outcome was the change in QTc interval (ΔQTcF, in milliseconds) from baseline to the first follow-up ECG, calculated using Fridericia’s correction formula, recommended by International Council for Harmonisation (ICH) E14 guidelines [19]. Bazett-corrected values (ΔQTcB) were computed for comparability with the Zhu et al. trial [6]. Secondary outcomes included newly emerged QTc prolongation (QTcF exceeding 450 ms in males or 470 ms in females among those with normal baseline); any QTc increase; clinically significant increase (exceeding 30 ms); severe QTc prolongation (QTcF exceeding 500 ms or increase exceeding 60 ms); and a composite cardiac safety endpoint comprising TdP, sustained ventricular tachycardia (VT), ventricular fibrillation (VF), cardiac arrest, or in-hospital death. Quality control procedures are described in Supplementary Method 2.

## Causal contrasts

We estimated two causal contrasts. The intention-to-treat (ITT) effect compared outcomes by initial statin assignment regardless of subsequent treatment changes. The per-protocol (PP) effect estimated the effect of sustained adherence; patients were censored at treatment deviation, with inverse probability of censoring weights (IPCW) applied [10] (Supplementary Method 4).

### Statistical analysis

A logistic regression model estimated the propensity score (PS), the probability of receiving rosuvastatin conditional on all measured baseline covariates [20]. The model included demographics, comorbidities, laboratory values, ECG and echocardiographic parameters, and concomitant medications (Supplementary Method 3). One-to-one nearest-neighbour PS matching was performed using a caliper of 0.2 standard deviations (SD) of the logit PS [20]. Covariate balance was assessed using absolute standardised mean differences (SMDs), with a threshold below 0.1 indicating adequate balance (Supplementary Fig. 2).

The primary ITT analysis used linear regression for ΔQTcF with robust standard errors, adjusting for residual imbalance in baseline QTcF, age, and sex. Modified Poisson regression with robust variance estimated risk ratios (RRs) for binary outcomes. The PP analysis incorporated IPCW with weights truncated at the 1st and 99th percentiles. Missing data were handled by complete-case analysis; patients with incomplete baseline covariates or missing ECG data were excluded at the eligibility stage (Fig. 1). Baseline covariate completeness exceeded 97% for all variables, and missingness was predominantly administrative rather than informative with respect to the outcome.

### Subgroup and sensitivity analyses

Pre-specified subgroup analyses examined effect heterogeneity by sex, age (below versus 65 years or above), heart failure, diabetes, chronic kidney disease (CKD), baseline QTcF, and beta-blocker use. Six sensitivity analyses were performed: Bazett correction; restriction to the 24–72 h window; PP analysis; exclusion of baseline QTc prolongation; E-value calculation [21]; and formal heterogeneity testing against the Zhu et al. RCT [6]. A negative control outcome analysis using headache (ICD-10: R51) was conducted to detect residual bias [22]. All analyses used R version 4.3. Two-sided P values below 0.05 were considered significant.

### Patient and public involvement

No patients or members of the public were involved in the design, conduct, reporting, or dissemination of this research.

## Results

### Study population

Of 619,216 cardiology hospitalizations between March 2012 and September 2024, 276,506 had suspected CAD, of whom 178,276 were confirmed new statin users after a 90-day look-back; following application of all protocol exclusions, most commonly incomplete data, 165,460 patients were eligible for TTE, comprising 85,789 rosuvastatin and 79,671 atorvastatin initiators (Fig. 1). After 1:1 PS matching, 98,860 patients (49,430 per group) constituted the analytic cohort, with excellent covariate balance (all SMDs below 0.013; Supplementary Fig. 1, Supplementary Fig. 2).

### Baseline characteristics

Table 2 presents the baseline characteristics. Mean age was 63.0 years (SD 12.0); 72% were male. Mean body mass index (BMI) was 24.2 kg/m² (SD 3.2). Hypertension was present in 60%, diabetes in 28%, heart failure in 5%. Mean baseline QTcF was 433.9 ms (SD 24.6); 7.3% had baseline QTc prolongation. Rosuvastatin was most commonly prescribed at 10 mg (85.0%), consistent with the moderate-intensity strategy recommended by Chinese lipid guidelines [8]. Atorvastatin was most commonly prescribed at 20 mg (80.0%). Notably, baseline QTc prolongation was present in 11.2% of rosuvastatin versus 11.7% of atorvastatin patients (SMD 0.016, *P* = 0.013); this was the smallest P value in Table 2, although the absolute difference was clinically trivial and the SMD remained well below the conventional threshold of 0.1, consistent with the known sensitivity of P values to sample size in large cohorts [23, 24]. The higher baseline rate in the atorvastatin group, if anything, biases the primary estimate toward the null. Our outcome model adjusted for baseline QTcF, providing doubly robust protection against this residual imbalance [25]. Dose distributions are detailed in Supplementary Table 6.

**Table 2:** Baseline characteristics of the post–propensity score matched cohort

Characteristic	Rosuvastatin (*n* = 49,430)	Atorvastatin (*n* = 49,430)	SMD	*P* value
Demographics	(*n* = 49,430)	(*n* = 49,430)		
Age, years, mean (SD)	63.0 (11.9)	63.0 (12.0)	0.000	0.945
Male, n (%)	13,743 (27.8)	14,020 (28.4)	0.012	0.051
Height, cm, mean (SD)	161.0 (7.7)	161.1 (7.8)	0.010	0.106
Weight, kg, mean (SD)	62.9 (10.3)	62.9 (10.3)	0.004	0.570
BMI, kg/m², mean (SD)	24.2 (3.2)	24.2 (3.2)	0.003	0.689
Current smoker, n (%)	11,447 (23.2)	11,382 (23.0)	0.008	0.283†
Former smoker, n (%)	2,762 (5.6)	2,876 (5.8)		
Never smoker, n (%)	35,221 (71.3)	35,172 (71.2)		
Current alcohol use, n (%)	5,840 (11.8)	5,834 (11.8)	0.005	0.625†
Former alcohol use, n (%)	2,402 (4.9)	2,468 (5.0)		
Comorbidities				
Hypertension, n (%)	29,645 (60.0)	29,697 (60.1)	0.002	0.741
Diabetes mellitus, n (%)	14,044 (28.4)	14,010 (28.3)	0.002	0.816
Heart failure, n (%)	2,375 (4.8)	2,446 (4.9)	0.007	0.301
Atrial fibrillation, n (%)	2,557 (5.2)	2,459 (5.0)	0.009	0.160
Chronic kidney disease, n (%)	3,523 (7.1)	3,452 (7.0)	0.006	0.385
Prior stroke/TIA, n (%)	6,995 (14.2)	6,825 (13.8)	0.010	0.121
Hypothyroidism, n (%)	4,895 (9.9)	4,915 (9.9)	0.001	0.840
Baseline laboratory values				
Serum potassium, mmol/L	4.1 (0.4)	4.1 (0.4)	0.003	0.646
Serum calcium, mmol/L	2.2 (0.1)	2.2 (0.1)	0.003	0.665
Serum magnesium, mmol/L	0.9 (0.1)	0.9 (0.1)	0.004	0.496
Serum sodium, mmol/L	139.9 (3.5)	139.9 (3.5)	0.004	0.485
Serum creatinine, µmol/L	76.7 (22.3)	76.8 (22.2)	0.002	0.793
eGFR, mL/min/1.73 m²	82.5 (22.3)	82.6 (22.3)	0.004	0.572
AST, U/L	29.5 (12.9)	29.4 (12.9)	0.003	0.665
ALT, U/L	27.6 (13.0)	27.6 (13.0)	0.004	0.570
Total cholesterol, mmol/L	4.5 (1.1)	4.5 (1.1)	0.004	0.483
LDL-C, mmol/L	2.9 (0.9)	2.9 (0.9)	0.000	0.966
HDL-C, mmol/L	1.2 (0.3)	1.2 (0.3)	0.007	0.244
Triglycerides, mmol/L	1.7 (0.9)	1.7 (0.9)	0.016	0.010
HbA1c, %	6.3 (1.3)	6.3 (1.3)	0.002	0.789
Baseline ECG parameters				
Heart rate, bpm	75.3 (13.9)	75.3 (13.9)	0.005	0.398
QTcF, ms	433.9 (23.7)	433.9 (23.8)	0.000	0.956
QTcB, ms	436.9 (24.2)	436.9 (24.3)	0.000	0.983
QT interval, ms	405.4 (34.1)	405.5 (34.4)	0.004	0.494
Baseline QTc prolongation, n (%)	5,517 (11.2)	5,765 (11.7)	0.016	0.013
Echocardiographic parameters				
LVEF, %	60.8 (7.4)	60.8 (7.4)	0.002	0.707
LVDd, mm	48.9 (6.2)	48.9 (6.2)	0.010	0.130
IVST, mm	10.7 (2.1)	10.7 (2.1)	0.013	0.045
LVPW, mm	10.1 (1.7)	10.1 (1.8)	0.001	0.852
Concomitant medications				
Aspirin, n (%)	44,494 (90.0)	44,558 (90.1)	0.004	0.503
Clopidogrel, n (%)	39,559 (80.0)	39,628 (80.2)	0.003	0.588
Ticagrelor, n (%)	1,481 (3.0)	1,475 (3.0)	0.001	0.926
Beta-blocker, n (%)	36,046 (72.9)	36,114 (73.1)	0.003	0.631
ACEI/ARB/ARNI, n (%)	36,871 (74.6)	36,952 (74.8)	0.004	0.558
CCB, n (%)	14,910 (30.2)	14,891 (30.1)	0.001	0.901
Loop diuretic, n (%)	7,403 (15.0)	7,565 (15.3)	0.009	0.153
PPI, n (%)	22,928 (46.4)	22,745 (46.0)	0.007	0.246
Nitrate, n (%)	19,658 (39.8)	19,788 (40.0)	0.005	0.402
Metformin, n (%)	9,023 (18.3)	9,201 (18.6)	0.009	0.147
Insulin, n (%)	4,182 (8.5)	4,229 (8.6)	0.003	0.600
SGLT-2 inhibitor, n (%)	2,316 (4.7)	2,339 (4.7)	0.002	0.741
Sedative/hypnotic, n (%)	6,877 (13.9)	7,059 (14.3)	0.011	0.098
Statin dose				
Rosuvastatin 5 mg, n (%)	4,952 (10.0)	—		
Rosuvastatin 10 mg, n (%)	41,919 (84.8)	—		
Rosuvastatin 20 mg, n (%)	2,559 (5.2)	—		
Atorvastatin 10 mg, n (%)	—	7,468 (15.1)		
Atorvastatin 20 mg, n (%)	—	39,480 (79.9)		
Atorvastatin 40 mg, n (%)	—	2,482 (5.0)		

Abbreviations: *ACEI*, angiotensin-converting enzyme inhibitor; *ARB*, angiotensin receptor blocker; *ARNI*, angiotensin receptor–neprilysin inhibitor; *CCB*, calcium channel blocker; *CKD*, chronic kidney disease; *eGFR*, estimated glomerular filtration rate; *IVST*, interventricular septal thickness; *LDL-C*, low-density lipoprotein cholesterol; *HDL-C*, high-density lipoprotein cholesterol; *LVEF*, left ventricular ejection fraction; *LVDd*, left ventricular end-diastolic dimension; *LVPW*, left ventricular posterior wall; *PPI*, proton pump inhibitor; *QTcB*, Bazett-corrected QT; *QTcF*, Fridericia-corrected QT; *SGLT-2*, sodium-glucose cotransporter 2; *SMD*, standardized mean difference; *TIA*, transient ischemic attack

Values are mean (SD) for continuous variables and n (%) for categorical variables unless otherwise specified

†Overall Pearson chi-square test for multi-level categorical variables

### Follow-up

Mean time from time zero to the follow-up ECG was 48.0 h (SD 10.0) in both groups (Supplementary Table 2). Most patients (93.5%) had their follow-up ECG within the primary 24–72 h window. Treatment deviation occurred in 8.0% of patients in each group. A total of 90,985 patients (92.0%) were eligible for the PP analysis.

### Primary outcome

In the ITT analysis, mean ΔQTcF was + 7.71 ms (SD 20.41) in the rosuvastatin group and + 0.31 ms (SD 22.30) in the atorvastatin group. The between-group mean difference was 7.40 ms (95% confidence interval [CI] 7.13 to 7.67; *P* < 0.001) (Table 3). ΔQTcB showed a consistent difference of 7.42 ms (95% CI 7.15 to 7.69; *P* < 0.001). Heart rate change did not differ between groups, suggesting the QTc difference was not an artefact of rate correction. The distribution of ΔQTcF is illustrated in Supplementary Fig. 3.

**Table 3:** Primary and secondary outcomes (intention-to-treat analysis)

Outcome	Rosuvastatin (*n* = 49,430)	Atorvastatin (*n* = 49,430)	Effect estimate	95% CI	*P* value
Primary outcome					
ΔQTcF, ms, mean (SD)	7.71 (20.41)	0.31 (22.30)	7.40	(7.13, 7.66)	0.00e + 00
ΔQTcB, ms, mean (SD)	8.70 (20.65)	1.28 (22.51)	7.42	(7.15, 7.69)	0.00e + 00
ΔHeart rate, bpm, mean (SD)	-3.96 (4.95)	-3.98 (4.95)	0.02	(-0.04, 0.08)	0.546
Secondary outcomes					
Newly emerged QTc prolongation	7,249 (14.7%)	5,180 (10.5%)	RR 1.40; RD 4.2%	RR (1.35, 1.45); RD (3.8%, 4.6%)	1.39e-87
Any QTc increase	32,001 (64.7%)	24,928 (50.4%)	RR 1.28; RD 14.3%	RR (1.27, 1.30); RD (13.7%, 14.9%)	0.00e + 00
Clinically significant increase (> 30 ms)	6,674 (13.5%)	4,506 (9.1%)	RR 1.48; RD 4.4%	RR (1.43, 1.53); RD (4.0%, 4.8%)	5.32e-105
Severe QTc prolongation	1,732 (3.5%)	1,217 (2.5%)	RR 1.42; RD 1.0%	RR (1.32, 1.53); RD (0.8%, 1.3%)	7.29e-22
Composite cardiac safety endpoint	200 (0.4%)	224 (0.5%)	RR 0.89; RD -0.0%	RR (0.74, 1.08); RD (-0.1%, 0.0%)	2.63e-01
Torsades de Pointes	4 (0.0%)	9 (0.0%)	RR 0.44; RD -0.0%	RR (0.14, 1.44); RD (-0.0%, 0.0%)	2.67e-01
Sustained ventricular tachycardia	49 (0.1%)	67 (0.1%)	RR 0.73; RD -0.0%	RR (0.51, 1.06); RD (-0.1%, 0.0%)	1.14e-01
Ventricular fibrillation	30 (0.1%)	22 (0.0%)	RR 1.36; RD 0.0%	RR (0.79, 2.36); RD (-0.0%, 0.0%)	3.32e-01
Cardiac arrest	14 (0.0%)	13 (0.0%)	RR 1.08; RD 0.0%	RR (0.51, 2.29); RD (-0.0%, 0.0%)	1.00e + 00
In-hospital death	104 (0.2%)	113 (0.2%)	RR 0.92; RD -0.0%	RR (0.71, 1.20); RD (-0.1%, 0.0%)	5.87e-01

Primary outcome: mean difference (rosuvastatin minus atorvastatin) from propensity score–matched linear regression with robust standard errors. Secondary binary outcomes: *RR* = risk ratio from modified Poisson regression; *RD* = risk difference from marginal standardization. *CI* = confidence interval. Newly emerged QTc prolongation defined as QTcF > 450 ms (males) or > 470 ms (females) among those with normal baseline QTcF. Severe QTc prolongation defined as QTcF > 500 ms or ΔQTcF > 60 ms

### Secondary outcomes

**Fig. 3:**
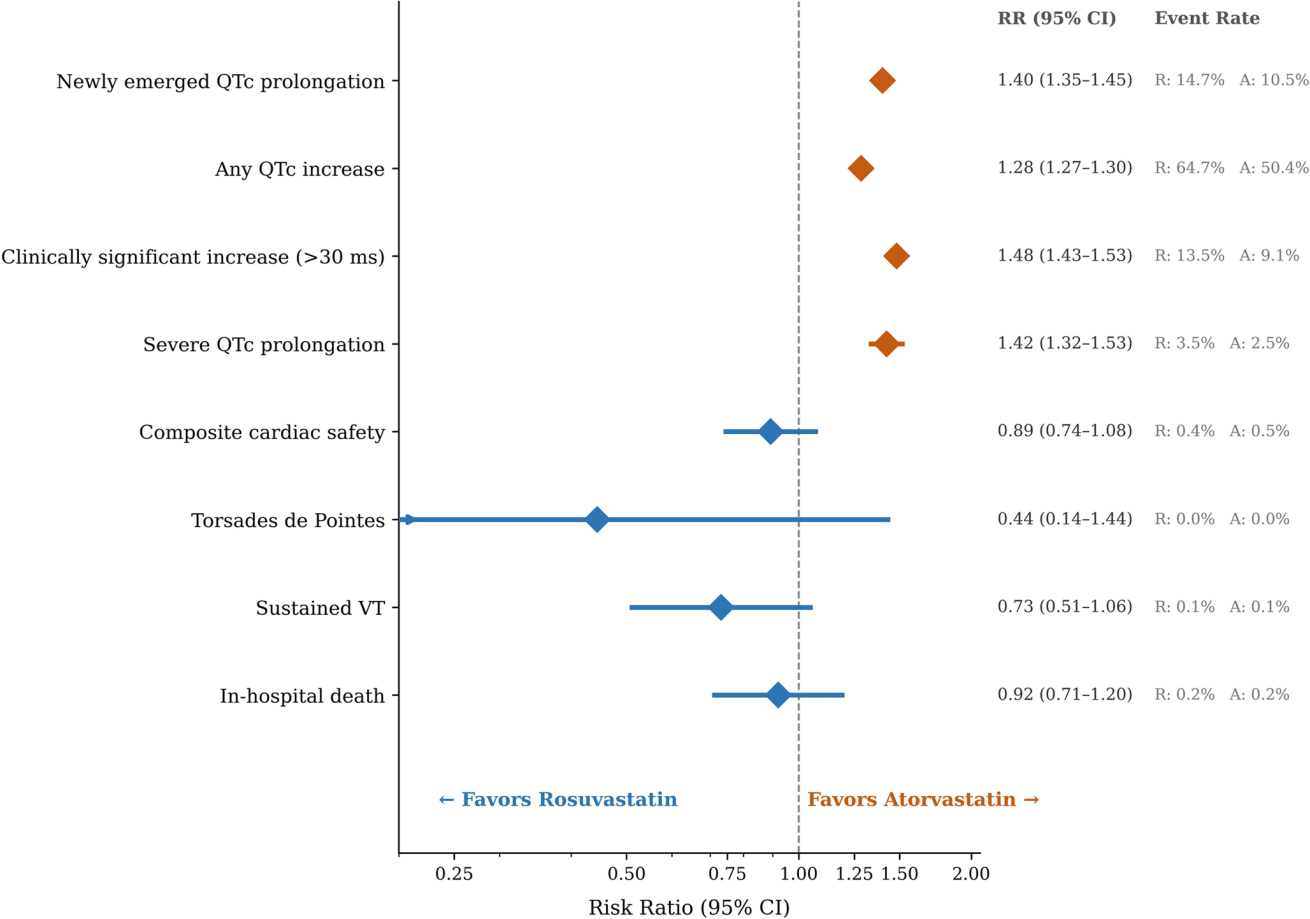
is the forest plot for secondary binary outcomes (ITT analysis), showing risk ratios with 95% CIs for newly emerged QTc prolongation (RR 1.40), any QTc increase (RR 1.28), clinically significant increase > 30 ms, severe QTc prolongation, composite cardiac safety, TdP, sustained VT, and in-hospital death. Event rates for each group are annotated alongside

Newly emerged QTc prolongation occurred in 14.7% of rosuvastatin versus 10.5% of atorvastatin patients (RR 1.40, 95% CI 1.35 to 1.45; *P* < 0.001) (Table 3; Fig. 3). Any QTc increase was observed in 64.7% versus 50.4% (RR 1.28, 95% CI 1.27 to 1.30). Clinically significant increase exceeding 30 ms occurred in 13.5% versus 9.1% (RR 1.48, 95% CI 1.43 to 1.53). Severe QTc prolongation was observed in 3.5% versus 2.5% (RR 1.42, 95% CI 1.32 to 1.53). The composite cardiac safety endpoint did not differ significantly (0.4% versus 0.5%; RR 0.89, 95% CI 0.74 to 1.08; *P* = 0.24). No significant differences were observed for TdP, sustained VT, or in-hospital death (Table 3). Event rates are illustrated in Supplementary Fig. 4.

### Per-protocol analysis

In the PP analysis (*n* = 90,985), the between-group mean difference in ΔQTcF was 7.38 ms (95% CI 7.10 to 7.66; *P* < 0.001), consistent with the ITT estimate (Table 4). PP estimates for secondary outcomes were similarly consistent.

**Table 4:** Per-protocol analysis results

Outcome	Rosuvastatin	Atorvastatin	Effect estimate	95% CI	*P* value
Per-protocol population	*n* = 45,461	*n* = 45,482			
ΔQTcF, ms, mean (SD)	7.68 (20.40)	0.30 (22.35)	7.38	(7.10, 7.66)	0.00e + 00
Newly emerged QTc prolongation	6,676 (14.7%)	4,802 (10.6%)	RR 1.39	(1.34, 1.44)	2.87e-78
Any QTc increase	29,427 (64.7%)	22,943 (50.4%)	RR 1.28	(1.27, 1.30)	0.00e + 00
Severe QTc prolongation	1,584 (3.5%)	1,120 (2.5%)	RR 1.41	(1.31, 1.53)	1.41e-19

Per-protocol population includes patients who adhered to assigned statin without discontinuation, switching, dose change > 50%, or initiation of QT-prolonging drugs through the follow-up ECG assessment. Inverse probability of censoring weights (IPCW) applied to adjust for informative censoring at treatment deviation. Weights truncated at 1st and 99th percentiles

### Subgroup analyses

**Fig. 4:**
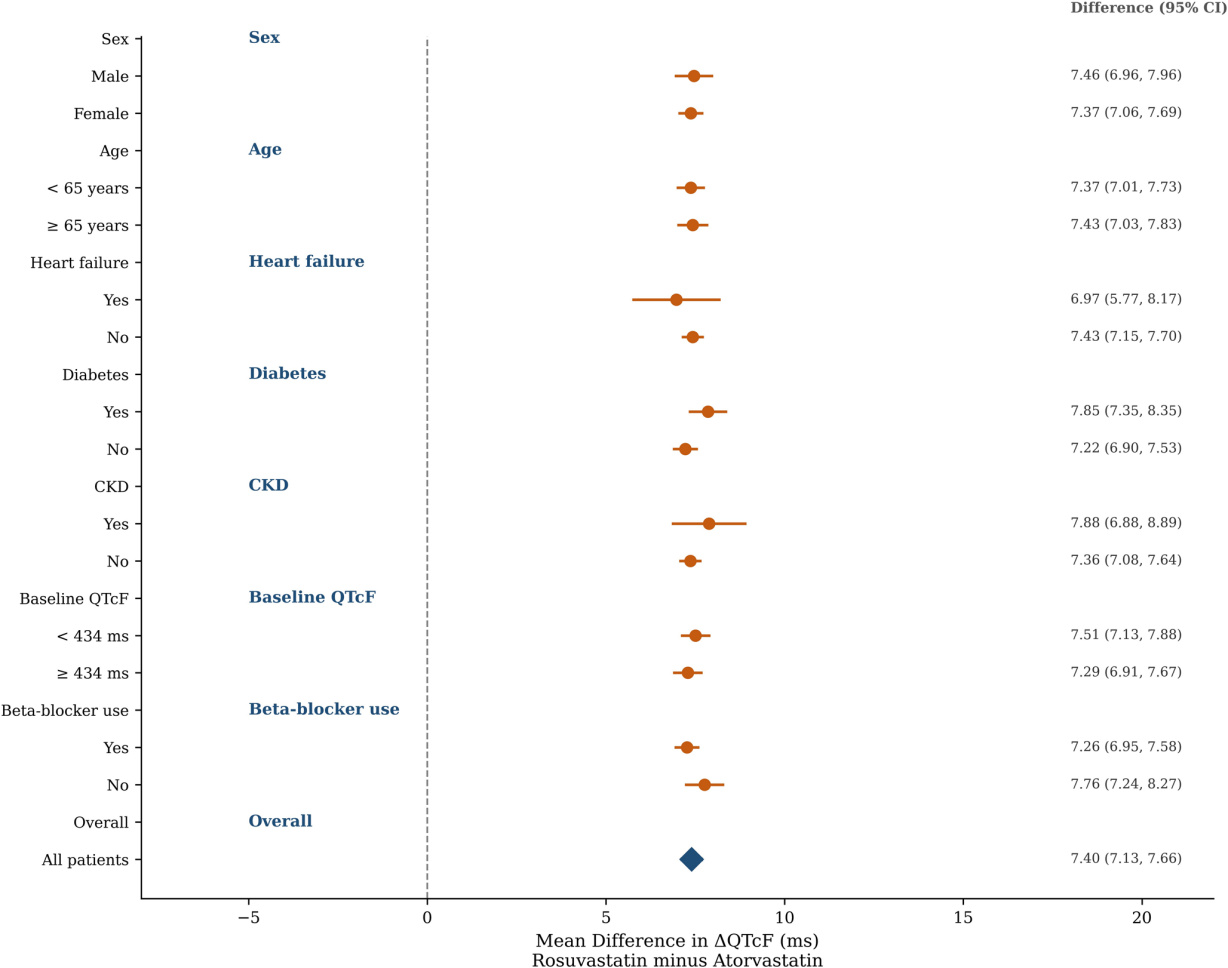
is the subgroup analysis forest plot showing the mean difference in ΔQTcF (rosuvastatin minus atorvastatin) across eight subgroup dimensions: sex, age, heart failure, diabetes, CKD, baseline QTcF, beta-blocker use, and the overall estimate. The rosuvastatin QTc-prolonging effect is consistent across all subgroups

The rosuvastatin-associated QTcF prolongation was consistent across all pre-specified subgroups (Fig. 4, Supplementary Table 3). The between-group difference ranged from 6.5 to 10.1 ms. No significant treatment-by-subgroup interactions were observed. The effect appeared numerically larger in patients with heart failure (10.1 ms) and CKD (9.2 ms), although these subgroup estimates had wider CIs.

### Sensitivity analyses

**Fig. 5:**
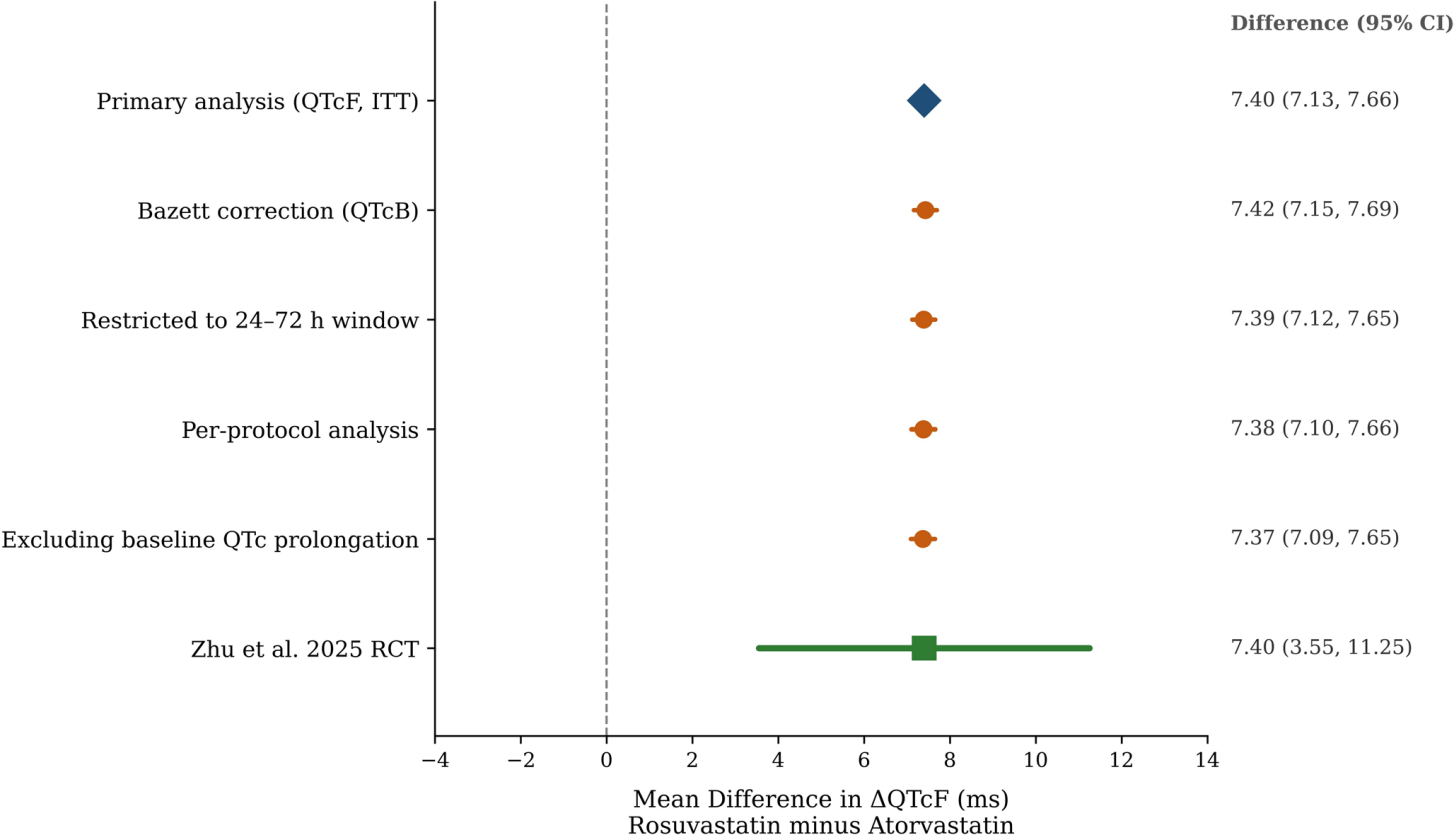
is the sensitivity analysis forest plot comparing the primary estimate against five alternative analytical approaches (Bazett correction, restricted time window, per-protocol, excluding baseline prolongation) plus the Zhu et al. RCT estimate. The TTE estimates are consistent across all approaches and show no significant heterogeneity with the published RCT (*P* = 0.923)

The primary finding was robust across all sensitivity analyses (Fig. 5, Supplementary Table 4). Bazett correction yielded 7.42 ms. Restriction to 24–72 h produced 7.39 ms. PP analysis estimated 7.38 ms. Exclusion of baseline QTc prolongation yielded 7.37 ms. The E-value for newly emerged prolongation (RR 1.40) was 2.14, indicating that an unmeasured confounder would need a risk ratio of at least 2.14-fold with both treatment and outcome to explain away the finding.

Completeness of baseline covariates in the eligibility-confirmed cohort is shown in Supplementary Table 7.

## Discussion

### Principal findings

In this TTE of nearly 100,000 propensity score–matched patients, rosuvastatin was associated with a mean QTcF prolongation of 7.40 ms compared with atorvastatin over the first 24–72 h of treatment. This estimate was virtually identical to the 7.40 ms difference reported in the Zhu et al. RCT of 466 patients [6], with a substantially narrower CI. Newly emerged QTc prolongation occurred in 14.7% of rosuvastatin patients versus 10.5% receiving atorvastatin. The effect was consistent across all pre-specified subgroups and robust to multiple sensitivity analyses. No significant between-group difference was observed in the composite cardiac safety endpoint.

### Comparison with existing evidence

Our findings align with a convergent body of preclinical and clinical evidence. Plante et al. showed that rosuvastatin blocks hERG current at clinically relevant concentrations [3]. Feng et al. identified additional intracellular mechanisms that reduce mature hERG protein expression [4]. Koo et al. reported an association between rosuvastatin and QT prolongation across over one million ECGs in a Korean database [5]. The Zhu et al. RCT provided the first randomized confirmation [6].

What distinguishes our study is scale. The original trial enrolled 466 patients. We analyzed 98,860. This 212-fold increase in sample size allowed detection of effects that a small trial simply cannot resolve. It enabled evaluation of eight subgroups, including heart failure, CKD, and diabetes, where the original trial had no statistical power. It also allowed assessment of rare safety outcomes such as TdP and cardiac arrest.

Reassuringly, the composite cardiac safety endpoint did not differ between groups. This is consistent with the meta-analysis by Rahimi et al. [26], which found that statins as a class modestly reduce sudden cardiac death without increasing ventricular arrhythmias. The rosuvastatin–QTc signal, while real, appears insufficient to translate into excess clinical events at the population level.

#### Implications of emulating a target trial at scale

The close concordance between our TTE estimate and the published RCT result carries important methodological implications. The RCT DUPLICATE initiative demonstrated that observational emulations can reproduce trial findings when design flaws are avoided [14]. Our study extends this validation to a short-term pharmacological safety outcome, a domain where TTE has rarely been tested.

The larger sample size offered three distinct advantages. First, precision. The 95% CI narrowed from 7.70 ms in the RCT to 0.54 ms in our study. This allows confident exclusion of trivially small effects. Second, generalizability. The Zhu et al. trial excluded patients on concomitant QT-prolonging medications, precisely the population at highest risk. Our cohort reflects routine clinical practice, including polypharmacy. Third, subgroup resolution. The consistent effect across sex, age, comorbidities, and medication categories suggests that rosuvastatin-induced QTc prolongation is not confined to a vulnerable subset. It is a pharmacological class effect of the drug itself. The close concordance was confirmed quantitatively: the TTE estimate (7.40 ms, 95% CI 7.13 to 7.67) showed no significant heterogeneity with the Zhu et al. RCT finding (7.40 ms, 95% CI 3.55 to 11.25; z = − 0.00, *P* = 1.00) (Supplementary Fig. 5, Supplementary Table 5). The negative control outcome analysis showed no association between rosuvastatin and headache (RR 1.02, 95% CI 0.94 to 1.10; *P* = 0.68), providing further reassurance against residual confounding.

These findings have practical implications. Clinicians should exercise caution when prescribing rosuvastatin to patients with pre-existing QTc prolongation, electrolyte disturbances, or concomitant use of other QT-prolonging agents. For most patients, the QTc effect is modest and does not outweigh the cardiovascular benefits of statin therapy. Atorvastatin remains a reasonable alternative where QTc risk is a concern.

#### Strengths and limitations

This study has several strengths. It is the largest investigation of rosuvastatin and QTc to date. The TTE framework ensured alignment of eligibility, treatment assignment, and follow-up at time zero, avoiding immortal time bias [10, 12]. The active comparator new-user design reduced confounding by indication [13]. PS matching achieved excellent covariate balance (all SMDs below 0.013). Multiple sensitivity analyses, including E-value, negative control, and formal heterogeneity testing against the RCT, provided robust evidence against residual confounding.

Several limitations should be acknowledged. First, this was a single-center study. The prescribing patterns and patient demographics reflect one Chinese tertiary hospital and may not generalize to other settings. Second, despite comprehensive PS adjustment, unmeasured confounding cannot be excluded. The E-value of 2.14 suggests that an unmeasured confounder would need to be moderately strong to explain away the finding. Third, we used Fridericia correction as the primary formula, while the Zhu et al. trial used Bazett correction. The sensitivity analysis with QTcB yielded nearly identical results (7.42 ms), confirming that the choice of correction formula did not influence conclusions. Fourth, our study assessed short-term effects only. Whether QTc prolongation persists, attenuates, or worsens with chronic rosuvastatin use remains unknown. Fifth, the data were collected from EHRs, which are subject to documentation variability, missing data, and potential coding errors. The most common exclusion reason was incomplete data, which could introduce selection bias if missingness is related to the outcome. Sixth, the mean follow-up of 48 h limits the interpretability of composite cardiac safety endpoints; hard clinical events such as TdP, sustained VT, VF, and cardiac arrest are rare within this timeframe, and the absence of a between-group difference should be interpreted with caution rather than as definitive evidence of safety [27]. Longer follow-up studies are needed to determine whether the observed QTc prolongation translates into excess clinical events with chronic rosuvastatin use.

## Conclusions

In this TTE of 98,860 propensity score–matched patients, rosuvastatin was associated with a 7.40 ms greater QTcF prolongation than atorvastatin, an effect that was consistent with the published RCT and stable across subgroups and analytical approaches. The absence of excess clinical cardiac events is reassuring. These findings support heightened electrocardiographic vigilance when prescribing rosuvastatin to patients at elevated risk for QTc prolongation, while confirming that the TTE framework can successfully replicate short-term pharmacological safety findings from randomized trials at substantially greater scale and precision.

## Supplementary Information

Below is the link to the electronic supplementary material.

Supplementary Material 1

## Data Availability

The de-identified analytic dataset and statistical analysis code supporting the findings of this study are available from the corresponding author upon reasonable request, subject to institutional data use agreements and approval from the participating hospitals’ data governance committees. Individual-level electronic health record data cannot be shared publicly due to patient privacy regulations and institutional data security policies.
