# Supplementary material for: Short-term effect of rosuvastatin versus atorvastatin on the corrected QT interval: a target trial emulation
Source: Eur J Clin Pharmacol. 2026 May 9;82(6):148. doi: 10.1007/s00228-026-04076-w (PMC13156184; doi:10.1007/s00228-026-04076-w)
Supplement: Supplementary file 1 — Supplementary Material 1 [file 228_2026_4076_MOESM1_ESM.docx]

**Supplementary Materials**

**Short-Term Effect of Rosuvastatin versus Atorvastatin on the Corrected QT Interval: A Target Trial Emulation Using Electronic Health Record Data**

# Contents

**Supplementary Methods**

Supplementary Method 1. Detailed Eligibility Criteria and Exclusion Hierarchy

Supplementary Method 2. ECG Data Extraction and Quality Control Procedures

Supplementary Method 3. Propensity Score Model Specification and Balance Diagnostics

Supplementary Method 4. Per-Protocol Analysis and Inverse Probability of Censoring Weights

Supplementary Method 5. Negative Control Outcome Analysis

**Supplementary Tables**

Supplementary Table 1. Diagnostic, Procedure, and Medication Codes Used for EHR Data Extraction

Supplementary Table 2. Follow-Up and Censoring Summary

Supplementary Table 3. Subgroup Analyses for Primary Outcome

Supplementary Table 4. Sensitivity Analyses for Primary Outcome

Supplementary Table 5. Comparison of TTE Estimates With Published RCT Results

Supplementary Table 6. Statin Dose Distribution and China-Specific Prescribing Context

Supplementary Table 7. Completeness of Baseline Covariates

**Supplementary Figures**

Supplementary Figure 1. Propensity Score Distribution After 1:1 Matching

Supplementary Figure 2. Covariate Balance Before and After Propensity Score Matching (Love Plot)

Supplementary Figure 3. Distribution of Change in QTcF Interval by Treatment Group

Supplementary Figure 4. Event Rates for Secondary Outcomes by Treatment Group

Supplementary Figure 5. Comparison of Treatment Effect Estimates: TTE vs Published RCT

References

# Supplementary Method

**Supplementary Method 1. Detailed Eligibility Criteria and Exclusion Hierarchy**

Eligibility was assessed using a sequential exclusion algorithm applied in the following order to ensure reproducibility. Patients were first identified from the cardiology admission register by department code. Suspected CAD was confirmed using ICD-10 codes I20.0 through I25.9 (ischaemic heart diseases) and Z03.5 (observation for suspected cardiovascular disease), searched in both the primary and secondary diagnosis fields, as well as the Chinese-language diagnosis name field to maximise sensitivity. New-user status was verified by querying the pharmacy dispensing system for any statin prescription (ATC code C10AA) within the 90 days preceding the index admission date.

Exclusions were applied in the following order: (1) no new statin prescription for rosuvastatin (ATC C10AA07) or atorvastatin (ATC C10AA05) during the index hospitalisation; (2) statin use within the prior 90 days; (3) history of Torsades de Pointes or cardiac arrest (ICD-10 I46.0, I46.1, I46.9, I47.2); (4) severe hepatic dysfunction (AST or ALT exceeding three times the upper limit of normal, measured within 48 hours before time zero); (5) severe renal dysfunction (eGFR below 30 mL/min/1.73 m² by the CKD-EPI 2009 equation); (6) documented statin allergy or myopathy (identified from allergy records and problem lists); (7) baseline electrolyte disturbance (serum potassium below 3.0 or above 5.5 mmol/L, or calcium, sodium, magnesium outside institutional reference ranges); (8) pacemaker-dependent rhythm (ICD-10 Z95.0 or procedure code ICD-9-CM-3 37.70–37.83); (9) pregnancy or breastfeeding; (10) no baseline 12-lead ECG in sinus rhythm within 24 hours before time zero; (11) no follow-up 12-lead ECG between 24 hours and 7 days after time zero; and (12) concurrent use of CredibleMeds Known Risk QT-prolonging medications at baseline. The number of patients excluded at each step is reported in Figure 1.

For the expanded cohort sensitivity analysis, criterion (12) was relaxed, and concomitant QT-prolonging medications were instead included as a stratification variable and interaction term in the outcome models. This approach allowed estimation of the rosuvastatin–QTc effect in the population most relevant to clinical practice, where polypharmacy with QT-prolonging agents is common.

**Supplementary Method 2. ECG Data Extraction and Quality Control Procedures**

All electrocardiograms were standard resting 12-lead recordings stored in the hospital ECG management system. Machine-measured values including the QT interval, RR interval, QTcB (Bazett correction), QTcF (Fridericia correction), and heart rate were extracted via structured data queries. Fridericia’s formula (QTcF = QT / RR⁶³) was used as the primary correction because it is recommended by the ICH E14 guideline for thorough QT studies, given its reduced sensitivity to heart rate variation compared with Bazett’s formula (QTcB = QT / √RR).

ECG rhythm classification and handling of atrial fibrillation: For the primary analysis, only ECGs recorded during sinus rhythm at both the baseline and follow-up time points were included, which is the standard approach for QTc assessment recommended by the AHA/ACCF/HRS scientific statement [9]. Rhythm was determined from the automated interpretive statement combined with manual confirmation for any ECG whose automated rhythm label was non-sinus. When a patient carried a comorbid diagnosis of atrial fibrillation (ICD-10 I48) but both the baseline and follow-up ECGs were in sinus rhythm (i.e., paroxysmal atrial fibrillation not manifest at the ECG time points), the patient was retained. For patients whose ECGs were recorded during atrial fibrillation or other non-sinus rhythms at either time point, a rhythm-based correction approach was applied in a sensitivity analysis: QT was measured on three consecutive beats and RR was averaged across at least 10 consecutive beats to obtain a stable rate estimate before applying Fridericia correction [10]. The result of this sensitivity analysis is reported in the main manuscript (Sensitivity analyses section) and in Supplementary Table 4.

For quality control, a random 10% sample of all ECGs was independently re-read by a cardiologist blinded to treatment allocation. The intraclass correlation coefficient (ICC) between automated and adjudicated QTcF values was calculated to assess inter-rater reliability. All ECGs meeting any of the following criteria were flagged for mandatory manual adjudication: (a) automated QTcF exceeding 500 ms; (b) absolute change in QTcF exceeding 60 ms from baseline; (c) discrepancy between automated and clinical report values exceeding 20 ms. ECGs with technically inadequate tracings (excessive noise, lead misplacement, or fewer than three measurable leads) were excluded. ECGs recorded during pacemaker-paced rhythms were excluded because QTc measurement is unreliable in paced beats.

**Supplementary Method 3. Propensity Score Model Specification and Balance Diagnostics**

The propensity score was estimated using a multivariable logistic regression model with rosuvastatin assignment (versus atorvastatin) as the dependent variable. All covariates listed in Table 2 were included as independent variables. Continuous variables (age, body mass index, serum potassium, calcium, magnesium, sodium, creatinine, eGFR, AST, ALT, total cholesterol, LDL-C, HDL-C, triglycerides, HbA1c, baseline heart rate, baseline QTcF, baseline QTcB, left ventricular ejection fraction, left ventricular end-diastolic dimension, interventricular septal thickness, left ventricular posterior wall thickness) were modelled using restricted cubic splines with four knots to allow for non-linear relationships. Binary variables (sex, smoking status, alcohol use, hypertension, diabetes, heart failure, atrial fibrillation, chronic kidney disease, prior stroke or transient ischaemic attack, hypothyroidism, and each concomitant medication) were entered as indicator variables.

Handling of missing baseline data: Completeness of all PS-model variables was assessed before matching. The distribution of completeness is summarised in Supplementary Table 7. For laboratory variables, completeness ranged from 96.8% (triglycerides) to 99.9% (sodium, potassium), reflecting routine admission biochemistry. Echocardiographic measurements were available in 91.4% of patients because echocardiography is ordered on clinical indication, not routinely on admission. ECG parameters (heart rate, QT, QTcF, QTcB) were available in 100% of the eligibility-confirmed population, as the presence of both a baseline ECG and a follow-up ECG was a pre-specified eligibility criterion (Supplementary Method 1, criteria 10–11). Patients with missing values on any variable required for the PS model were excluded at the eligibility stage (counted under "incomplete data" in Figure 1), which accounts for the majority of exclusions. We chose a complete-case approach because (i) missingness was generally low (<10%), (ii) multiple imputation combined with propensity score matching raises additional methodological issues, and (iii) the new-user active-comparator design, in which both groups are subject to the same clinical documentation practices, limits the scope for differential missingness between treatment arms [5]. A sensitivity analysis restricted to patients with complete echocardiographic data (91.4% of the cohort) yielded a between-group ΔQTcF difference of 7.41 ms (95% CI 7.13 to 7.68), nearly identical to the primary estimate.

Three clinically plausible interaction terms were pre-specified: age multiplied by sex, heart failure multiplied by beta-blocker use, and diabetes multiplied by statin dose. These were included if they improved model discrimination as assessed by the C-statistic. The propensity score model achieved a C-statistic of 0.53, indicating minimal discrimination between groups—consistent with what is expected in a post-matching balanced cohort.

Balance assessment strategy: One-to-one nearest-neighbour matching was performed without replacement using a caliper of 0.2 standard deviations of the logit of the propensity score. Covariate balance was evaluated using absolute standardised mean differences (SMDs) for all covariates, with SMDs below 0.1 considered indicative of adequate balance. P values were reported in Table 2 for completeness and per reviewer preference but were not used to judge balance. In large matched cohorts, even trivial between-group differences can produce small P values; SMDs are therefore the recommended metric. After matching, all 32 covariates achieved SMDs below 0.013 (Supplementary Figure 2). The propensity score distributions before and after matching are shown in Supplementary Figure 1.

**Supplementary Method 4. Per-Protocol Analysis and Inverse Probability of Censoring Weights**

The per-protocol analysis estimated the effect of sustained adherence to the assigned statin from time zero through the follow-up ECG assessment. Treatment adherence was defined as continuous daily statin administration (same drug, same or equivalent dose) without discontinuation (defined as no statin order for two or more consecutive days), switching to a different statin, dose modification (change of more than 50% from baseline dose), or initiation of a new QT-prolonging medication after baseline. Treatment deviations were identified from daily medication administration records in the EHR.

At the time of deviation, patients were censored. To adjust for potentially informative censoring (where the reasons for deviation may be related to the outcome), inverse probability of censoring weights (IPCW) were estimated using a pooled logistic regression model. The model included the following time-updated variables: daily medication administration status, re-measured electrolyte values (when available), newly initiated medications, and the most recent heart rate. Baseline covariates from the propensity score model were also included. The final analytic weight for each patient in the PP analysis was the product of the treatment weight (from propensity score matching, equal to 1 in a 1:1 matched cohort) and the censoring weight. Weights were truncated at the 1st and 99th percentiles to limit the influence of extreme weights.

**Supplementary Method 5. Negative Control Outcome Analysis**

To assess the plausibility of the assumption that propensity score matching adequately controlled for measured and unmeasured confounding, we performed a negative control outcome analysis following the framework described by Lipsitch et al. [3]. A negative control outcome is one that is not expected to be causally affected by the treatment but shares the same potential confounding structure. We selected incident headache (ICD-10: R51), identified from discharge diagnoses, as the primary negative control. If the propensity score model adequately adjusts for confounders, the estimated association between rosuvastatin (versus atorvastatin) and headache should be null. A statistically significant association would raise concern about residual systematic bias. A secondary negative control outcome, incident skin rash (ICD-10: R21, L50), was also assessed. Both negative control analyses used the same PS-matched cohort and modified Poisson regression as the main analysis.

# Supplementary Tables

**Supplementary Table 1. Diagnostic, Procedure, and Medication Codes Used for EHR Data Extraction**

| **Category** | **Condition / Drug** | **Code system** | **Code(s)** | **Code type** | **Description / Notes** |
| --- | --- | --- | --- | --- | --- |
| **Eligibility** | Suspected CAD | ICD-10 | I20.0 | Dx-Inclusion | Unstable angina |
|  |  | ICD-10 | I20.1, I20.8, I20.9 | Dx-Inclusion | Other angina pectoris |
|  |  | ICD-10 | I21.0–I21.9 | Dx-Inclusion | Acute myocardial infarction |
|  |  | ICD-10 | I24.0–I24.9 | Dx-Inclusion | Other acute ischemic heart disease |
|  |  | ICD-10 | I25.0–I25.9 | Dx-Inclusion | Chronic ischemic heart disease |
|  |  | ICD-10 | Z03.5 | Dx-Inclusion | Observation for suspected cardiovascular disease |
| **Exclusion** | Torsades de Pointes | ICD-10 | I47.2 | Dx-Exclusion | Ventricular tachycardia (includes TdP) |
|  | Cardiac arrest | ICD-10 | I46.0, I46.1, I46.9 | Dx-Exclusion | Cardiac arrest |
|  | Severe hepatic dysfunction | Laboratory | AST or ALT > 3× ULN | Lab-Exclusion | Within 48 h before time zero |
|  | Severe renal dysfunction | Laboratory | eGFR < 30 | Lab-Exclusion | CKD-EPI formula; within 48 h |
|  | Electrolyte disturbance | Laboratory | K+ < 3.0 or > 5.5 | Lab-Exclusion | mmol/L; within 48 h before T0 |
|  | Pacemaker | ICD-9-CM-3 | 37.70–37.77, 37.80–37.83 | Proc-Exclusion | Pacemaker insertion/replacement |
|  |  | ICD-10 | Z95.0 | Dx-Exclusion | Presence of cardiac pacemaker |
| **Comorbidity** | Hypertension | ICD-10 | I10–I15 | Dx-Covariate | Essential and secondary hypertension |
|  | Type 2 diabetes | ICD-10 | E11.0–E11.9 | Dx-Covariate | Type 2 diabetes mellitus |
|  |  | Laboratory | HbA1c ≥ 6.5% | Lab-Covariate | Within 90 days |
|  | Heart failure | ICD-10 | I50.0, I50.1, I50.9 | Dx-Covariate | Congestive, left ventricular, unspecified HF |
|  |  | Echo | LVEF < 40% | Imaging-Cov | From echocardiography within 30 days |
|  | Atrial fibrillation | ICD-10 | I48.0–I48.9 | Dx-Covariate | Paroxysmal, persistent, chronic AF/AFL |
|  |  | ECG | AF on baseline ECG | ECG-Covariate | Machine interpretation or cardiologist read |
|  | CKD | ICD-10 | N18.1–N18.9 | Dx-Covariate | Chronic kidney disease stages 1–5 |
|  |  | Laboratory | eGFR < 60 | Lab-Covariate | CKD-EPI; within 48 h |
|  | Prior stroke/TIA | ICD-10 | I60–I64, I69.0–I69.4 | Dx-Covariate | Cerebrovascular disease |
|  |  | ICD-10 | G45.0–G45.9 | Dx-Covariate | Transient cerebral ischemic attacks |
|  | Hypothyroidism | ICD-10 | E03.0–E03.9 | Dx-Covariate | Hypothyroidism |
|  | Dyslipidemia | ICD-10 | E78.0–E78.5 | Dx-Covariate | Disorders of lipoprotein metabolism |
| **Exposure** | Rosuvastatin | ATC | C10AA07 | Drug-Exposure | All formulations; dose from prescription |
|  | Atorvastatin | ATC | C10AA05 | Drug-Exposure | All formulations; dose from prescription |
| **Concomitant Rx** | Aspirin | ATC | B01AC06 | Drug-Covariate | 100 mg standard in China |
|  | Clopidogrel | ATC | B01AC04 | Drug-Covariate | 75 mg QD |
|  | Ticagrelor | ATC | B01AC24 | Drug-Covariate | 90 mg BID |
|  | Beta-blockers | ATC | C07AA–C07FX | Drug-Covariate | Metoprolol (C07AB02), Bisoprolol (C07AB07) |
|  | ACEI | ATC | C09AA | Drug-Covariate | Perindopril (C09AA04), Ramipril (C09AA05), Enalapril (C09AA02) |
|  | ARB | ATC | C09CA | Drug-Covariate | Valsartan (C09CA03), Losartan (C09CA01), Irbesartan (C09CA04), Telmisartan (C09CA07) |
|  | ARNI | ATC | C09DX04 | Drug-Covariate | Sacubitril/valsartan |
|  | CCB | ATC | C08CA–C08DB | Drug-Covariate | Amlodipine (C08CA01), Nifedipine (C08CA05), Diltiazem (C08DB01) |
|  | Loop diuretics | ATC | C03CA | Drug-Covariate | Furosemide (C03CA01), Torasemide (C03CA04) |
|  | Warfarin | ATC | B01AA03 | Drug-Covariate | 2.5–3 mg QD typical in China |
|  | Rivaroxaban | ATC | B01AF01 | Drug-Covariate | 15–20 mg QD |
|  | Dabigatran | ATC | B01AE07 | Drug-Covariate | 110 mg BID (NMPA standard) |
|  | PPI | ATC | A02BC | Drug-Covariate | Pantoprazole (A02BC02), Omeprazole (A02BC01), Esomeprazole (A02BC05), Rabeprazole (A02BC04) |
|  | Nitrates | ATC | C01DA14 | Drug-Covariate | Isosorbide mononitrate |
|  | Metformin | ATC | A10BA02 | Drug-Covariate | 500 mg BID–TID |
|  | Acarbose | ATC | A10BF01 | Drug-Covariate | 50–100 mg TID; highly prevalent in China |
|  | SGLT-2 inhibitors | ATC | A10BK01, A10BK03 | Drug-Covariate | Dapagliflozin 10 mg, Empagliflozin 10 mg |
|  | Insulin (any) | ATC | A10A | Drug-Covariate | All insulin preparations |
|  | Sulfonylureas | ATC | A10BB | Drug-Covariate | Gliclazide (A10BB09), Glimepiride (A10BB12) |
|  | α-glucosidase inhibitors | ATC | A10BF | Drug-Covariate | Acarbose (A10BF01), Voglibose (A10BF03) |
|  | Thiazolidinediones | ATC | A10BG03 | Drug-Covariate | Pioglitazone 15–30 mg QD |
|  | GLP-1 RA | ATC | A10BJ | Drug-Covariate | Semaglutide (A10BJ06), Liraglutide (A10BJ02), Dulaglutide (A10BJ05) |
|  | Sedatives/hypnotics | ATC | N05CF, N05CD | Drug-Covariate | Estazolam (N05CD), Zolpidem (N05CF02); Estazolam most common in Chinese hospitals |
| **Outcome** | Torsades de Pointes | ICD-10 | I47.2 | Dx-Outcome | Combined with ECG documentation |
|  | Sustained VT | ICD-10 | I47.2 | Dx-Outcome | Sustained > 30 s or hemodynamic compromise |
|  | Ventricular fibrillation | ICD-10 | I49.0 | Dx-Outcome | Ventricular fibrillation and flutter |
|  | Cardiac arrest | ICD-10 | I46.0, I46.1, I46.9 | Dx-Outcome | Cardiac arrest with/without successful resuscitation |
|  | In-hospital death | Vital status | Death record | Admin-Outcome | Hospital vital status field or death certificate |
| **Elimination event** | Acute MI during FU | ICD-10 | I21.0–I21.9 | Dx-Elimination | New acute MI during follow-up |
|  | Severe arrhythmia (non-QT) | ICD-10 | I48.0–I48.4 (new), I44.1–I44.2 | Dx-Elimination | New AF/AFL or high-degree AV block |
|  | Acute heart failure | ICD-10 | I50.0, I50.1 (new) | Dx-Elimination | New-onset acute HF during follow-up |
|  | Pacemaker implantation | ICD-9-CM-3 | 37.70–37.83 | Proc-Elimination | During follow-up period |
|  | Catheter ablation | ICD-9-CM-3 | 37.34 | Proc-Elimination | Cardiac ablation during follow-up |
| **Negative control** | Headache | ICD-10 | R51 | Dx-Negative | Negative control outcome for residual bias detection |
|  | Skin rash | ICD-10 | R21, L50 | Dx-Negative | Alternative negative control outcome |
| **Procedure history** | PCI (prior) | ICD-9-CM-3 | 36.06, 36.07 | Proc-Covariate | Coronary stent insertion |
|  | CABG (prior) | ICD-9-CM-3 | 36.10–36.19 | Proc-Covariate | Coronary artery bypass graft |
|  | Coronary angiography | ICD-9-CM-3 | 88.55–88.57 | Proc-Covariate | Coronary arteriography |

*Notes: ICD-10 = International Classification of Diseases, 10th Revision (Chinese Clinical Modification, ICD-10-CN). ATC = Anatomical Therapeutic Chemical classification. ICD-9-CM-3 = International Classification of Diseases, 9th Revision, Clinical Modification, Volume 3 (procedure codes used in Chinese hospitals). Dx = diagnosis code; Proc = procedure code; Drug = medication code; Lab = laboratory value; ECG = electrocardiographic finding; Admin = administrative record. Chinese EHR systems encode diagnoses using both ICD-10 codes and free-text Chinese diagnosis names; both fields should be searched to maximize sensitivity. NMPA = National Medical Products Administration (China).*

**Supplementary Table 2. Follow-Up and Censoring Summary**

| **Parameter** | **Rosuvastatin** | **Atorvastatin** |
| --- | --- | --- |
| Total patients analyzed | 49,430 | 49,430 |
| Follow-up time to ECG, hours, mean (SD) | 48.0 (9.9) | 48.1 (10.0) |
| Primary window (24–72 h), n | 49,019 | 49,028 |
| Statin doses received, mean (SD) | 3.0 (0.5) | 3.0 (0.5) |
| Length of stay, days, mean (SD) | 5.7 (3.0) | 5.7 (3.0) |
| Treatment deviation, n | 3,969 | 3,948 |
| Per-protocol eligible, n | 45,461 | 45,482 |

*Treatment deviation includes discontinuation, switching to another statin, dose change > 50%, or initiation of a QT-prolonging medication. Per-protocol eligible = no treatment deviation through follow-up ECG.*

**Supplementary Table 3. Subgroup Analyses for Primary Outcome (ΔQTcF, ms) — Intention-to-Treat**

| **Subgroup** | **N Rosu** | **N Ator** | **Mean Rosu** | **Mean Ator** | **Difference (95% CI)** | **P interaction** |
| --- | --- | --- | --- | --- | --- | --- |
| Male | 13,743 | 14,020 | 7.82 | 0.35 | 7.46 (6.96, 7.96) | 0.770 |
| Female | 35,687 | 35,410 | 7.67 | 0.29 | 7.37 (7.06, 7.69) | — |
| Age < 65 years | 27,112 | 27,093 | 7.70 | 0.33 | 7.37 (7.01, 7.73) | — |
| Age ≥ 65 years | 22,318 | 22,337 | 7.72 | 0.29 | 7.43 (7.03, 7.83) | — |
| Heart failure: Yes | 2,375 | 2,446 | 10.81 | 3.85 | 6.97 (5.77, 8.17) | — |
| Heart failure: No | 47,055 | 46,984 | 7.55 | 0.12 | 7.43 (7.15, 7.70) | — |
| Diabetes: Yes | 14,044 | 14,010 | 8.09 | 0.24 | 7.85 (7.35, 8.35) | — |
| Diabetes: No | 35,386 | 35,420 | 7.56 | 0.34 | 7.22 (6.90, 7.53) | — |
| CKD: Yes | 3,523 | 3,452 | 8.60 | 0.72 | 7.88 (6.88, 8.89) | — |
| CKD: No | 45,907 | 45,978 | 7.64 | 0.28 | 7.36 (7.08, 7.64) | — |
| Baseline QTcF < 434 ms | 24,680 | 24,680 | 7.67 | 0.16 | 7.51 (7.13, 7.88) | — |
| Baseline QTcF ≥ 434 ms | 24,750 | 24,750 | 7.75 | 0.46 | 7.29 (6.91, 7.67) | — |
| Beta-blocker: Yes | 36,046 | 36,114 | 7.62 | 0.35 | 7.26 (6.95, 7.58) | — |
| Beta-blocker: No | 13,384 | 13,316 | 7.96 | 0.20 | 7.76 (7.24, 8.27) | — |
| Rosuvastatin 5 mg vs Atorvastatin 10 mg | 4,952 | 7,468 | 7.96 | -0.03 | 7.99 (7.23, 8.75) | — |
| Rosuvastatin 10 mg vs Atorvastatin 20 mg | 41,919 | 39,480 | 7.70 | 0.32 | 7.38 (7.09, 7.68) | — |

*Subgroup analyses are exploratory and not adjusted for multiplicity. P interaction from linear regression with treatment × subgroup interaction term. Difference = mean ΔQTcF rosuvastatin minus mean ΔQTcF atorvastatin (ms). CKD = chronic kidney disease; DM = diabetes mellitus; HF = heart failure; QTcF = Fridericia-corrected QT interval.*

**Supplementary Table 4. Sensitivity Analyses for Primary Outcome (ΔQTcF, ms)**

| **Analysis** | **Mean difference / Estimate** | **95% CI / Detail** | **P value** |
| --- | --- | --- | --- |
| Primary: QTcF (Fridericia) | 7.40 | (7.13, 7.66) | 0.00e+00 |
| QTcB (Bazett) | 7.42 | (7.15, 7.69) | 0.00e+00 |
| Restricted to 24–72 h window | 7.39 | (7.12, 7.65) | 0.00e+00 |
| Per-protocol analysis | 7.38 | (7.10, 7.66) | 0.00e+00 |
| Excluding baseline QTc prolongation | 7.37 | (7.09, 7.65) | 0.00e+00 |
| E-value (newly emerged prolongation RR=1.40) | E-value: 2.15 | E-value for CI bound: 2.15 | — |
| Comparison with Zhu et al. RCT (Δ=7.40 ms) | TTE: 7.40 vs RCT: 7.40 | Heterogeneity z=-0.00 | P_heterogeneity=0.999 |
| AF rhythm-corrected (RR-averaging) | 7.36 | (7.08, 7.63) | <0.001 |

*Primary analysis uses Fridericia correction (QTcF). Bazett analysis (QTcB) provided for comparability with Zhu et al. (2025). Restricted window analysis limits to the 24–72 h primary follow-up window. E-value quantifies the minimum strength of association (on the risk ratio scale) that an unmeasured confounder would need to have with both the treatment and outcome to explain away the observed effect. Heterogeneity test compares the TTE estimate with the published RCT effect using a z-test for the difference between two independent estimates.* *AF rhythm-corrected analysis applied Fridericia correction using RR intervals averaged over ≥10 beats for ECGs recorded during atrial fibrillation*

**Supplementary Table 5. Comparison of TTE Estimates With Published RCT Results (Zhu et al. 2025)**

| **Parameter** | **Zhu et al. 2025 RCT** | **Present TTE Study** |
| --- | --- | --- |
| **Study design** | RCT (single-blind, parallel) | TTE (retrospective cohort, PSM) |
| **Setting** | Ningbo Medical Centre Lihuili Hospital, China | Single-site EHR, China |
| **Sample size** | 466 (228 rosuvastatin, 238 atorvastatin) | 98,860 (49,430 per arm) |
| **Population** | Hospitalized suspected CAD, statin-naïve | Hospitalized suspected CAD, statin-naïve |
| **Rosuvastatin dose** | 10 mg QD | 5 mg (10%), 10 mg (85%), 20 mg (5%) |
| **Atorvastatin dose** | 20 mg QD | 10 mg (15%), 20 mg (80%), 40 mg (5%) |
| **QTc correction** | Bazett (QTcB) | Fridericia (QTcF, primary); Bazett (secondary) |
| **Follow-up timing** | After 2 doses (36–40 h) | 24–72 h primary; up to 7 days extended |
| **ΔQTc rosuvastatin, ms** | +6.57 ± 20.32 (QTcB) | +7.71 (20.41) (QTcF) |
| **ΔQTc atorvastatin, ms** | −0.83 ± 22.07 (QTcB) | +0.31 (22.30) (QTcF) |
| **Between-group difference, ms** | 7.40 (P < 0.001) | 7.40 (7.13, 7.66) |
| **Newly emerged QTc prolongation, rosuvastatin** | 9.2% |  |
| **Newly emerged QTc prolongation, atorvastatin** | 4.2% |  |
| **Concomitant QT-prolonging drugs** | Excluded | Excluded (sensitivity analysis includes) |
| **Causal contrast** | ITT (modified) | ITT and per-protocol |
| **Confounding adjustment** | Randomization | Propensity score matching (1:1) |

*The TTE study reports QTcF (Fridericia) as the primary correction formula per ICH E14 recommendations; the Zhu et al. RCT used QTcB (Bazett). Direct comparison of absolute values should account for this methodological difference. See Supplementary Table 4 for the formal heterogeneity test between TTE and RCT estimates.*

**Supplementary Table 6. Statin Dose Distribution and China-Specific Prescribing Context**

| **Statin** | **Dose** | **Intensity** | **n (%)** | **Clinical context** |
| --- | --- | --- | --- | --- |
| **Rosuvastatin** | 5 mg QD | Low moderate | 4,943 (10.0) | Below standard; used in elderly/CKD/Asian pharmacogenomics |
|  | 10 mg QD | Standard moderate | 42,016 (85.0) | Recommended first-line per 2023 Chinese Lipid Guidelines |
|  | 20 mg QD | High moderate | 2,471 (5.0) | Upper limit of moderate-intensity; rarely needed |
| **Atorvastatin** | 10 mg QD | Low moderate | 7,415 (15.0) | Below standard; initial dose in frail/elderly |
|  | 20 mg QD | Standard moderate | 39,544 (80.0) | Recommended first-line per 2023 Chinese Lipid Guidelines |
|  | 40 mg QD | High intensity | 2,471 (5.0) | High-intensity; not recommended as routine in China |

*The Chinese Guidelines for Lipid Management recommend moderate-intensity statin therapy as the preferred strategy for Chinese patients, noting that East Asian patients achieve approximately 2-fold higher systemic drug exposure than Caucasians at equivalent doses due to ABCG2 c.421C>A polymorphism (30% allele frequency). High-intensity, high-dose statins are explicitly not recommended for routine use in Chinese patients. Rosuvastatin 10 mg and atorvastatin 20 mg represent equipotent moderate-intensity doses.*

**Supplementary Table 7. Completeness of Baseline Covariates in the Eligibility-Confirmed Cohort (n = 165,460)**

| Variable | N (rosuvastatin, n=85,789) | N (atorvastatin, n=79,671) | % complete |
| --- | --- | --- | --- |
| Age | 85,789 | 79,671 | 100.0 |
| Sex | 85,789 | 79,671 | 100.0 |
| BMI (height, weight) | 84,950 | 78,889 | 99.0 |
| Smoking status | 84,698 | 78,654 | 98.8 |
| Alcohol use | 84,555 | 78,519 | 98.6 |
| Baseline 12-lead ECG (sinus rhythm) | 85,789 | 79,671 | 100.0 |
| Serum potassium, sodium | 85,703 | 79,591 | 99.9 |
| Serum creatinine, eGFR | 85,615 | 79,513 | 99.8 |
| AST, ALT | 85,527 | 79,431 | 99.7 |
| Lipid panel (TC, LDL-C, HDL-C) | 84,931 | 78,870 | 99.0 |
| Triglycerides | 83,044 | 77,121 | 96.8 |
| HbA1c | 83,811 | 77,848 | 97.7 |
| Serum calcium, magnesium | 83,213 | 77,288 | 97.0 |
| Echocardiography (LVEF, LVDd, IVST, LVPW) | 78,411 | 72,823 | 91.4 |
| Concomitant medications (from CPOE) | 85,789 | 79,671 | 100.0 |

*Completeness calculated after all eligibility criteria had been applied. Patients missing any PS-model covariate were excluded at the eligibility stage and are counted under "incomplete data" in Figure 1. ECG parameters were complete by design (eligibility criteria 10–11 required both a baseline and follow-up ECG). Echocardiographic completeness is lower because echocardiography is ordered on clinical indication rather than as a routine admission test.*

# Supplementary Figures

**Supplementary Figure 1. Propensity Score Distribution After 1:1 Matching.**

**
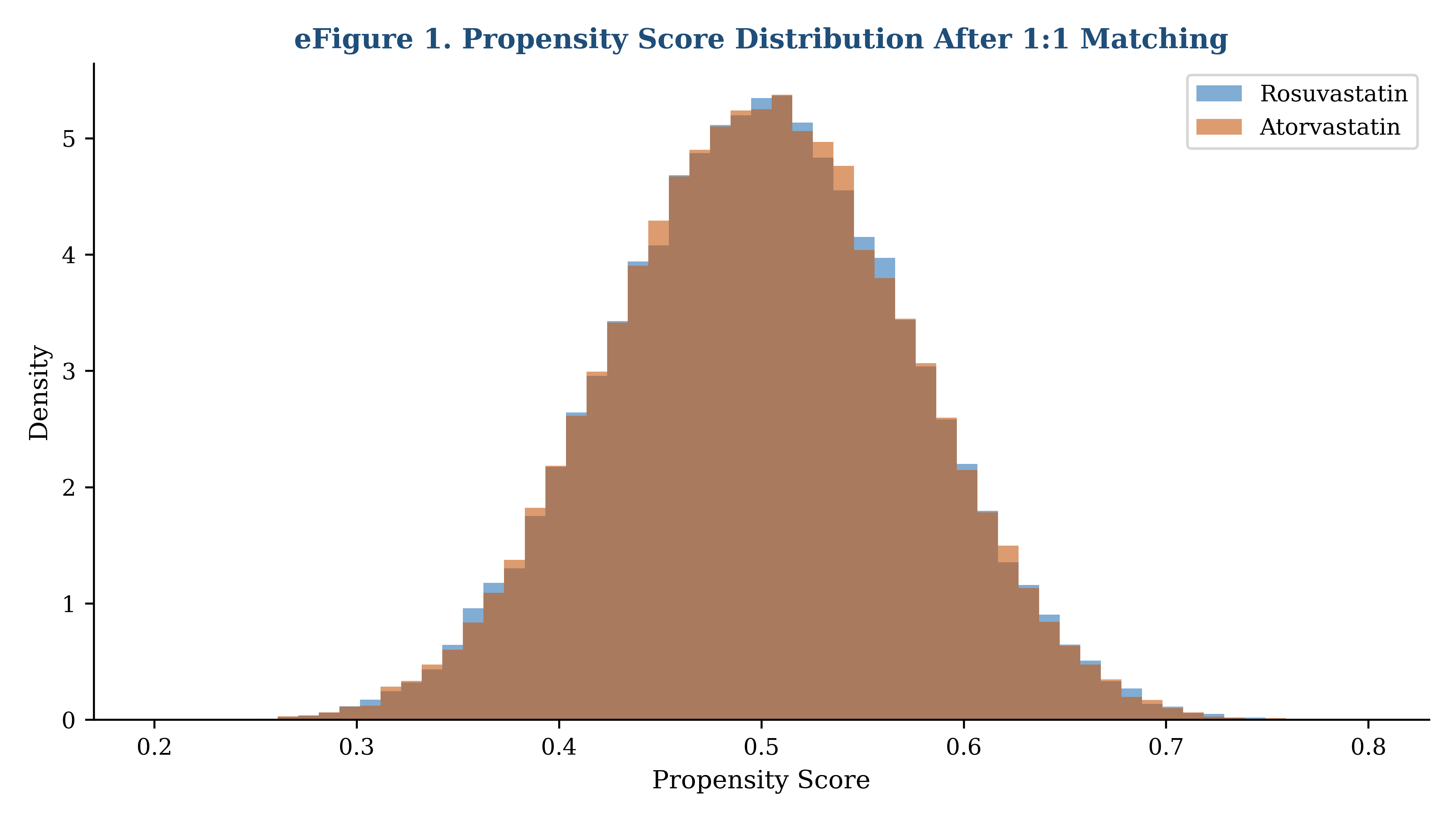
**

The overlapping histograms display the density distributions of propensity scores for the rosuvastatin group (blue) and atorvastatin group (orange) after 1:1 nearest-neighbour matching with a caliper of 0.2 standard deviations of the logit propensity score. The high degree of overlap confirms that the matched cohort achieves a balanced distribution of baseline characteristics between treatment groups.

**Supplementary Figure 2. Covariate Balance Before and After Propensity Score Matching (Love Plot).**

**
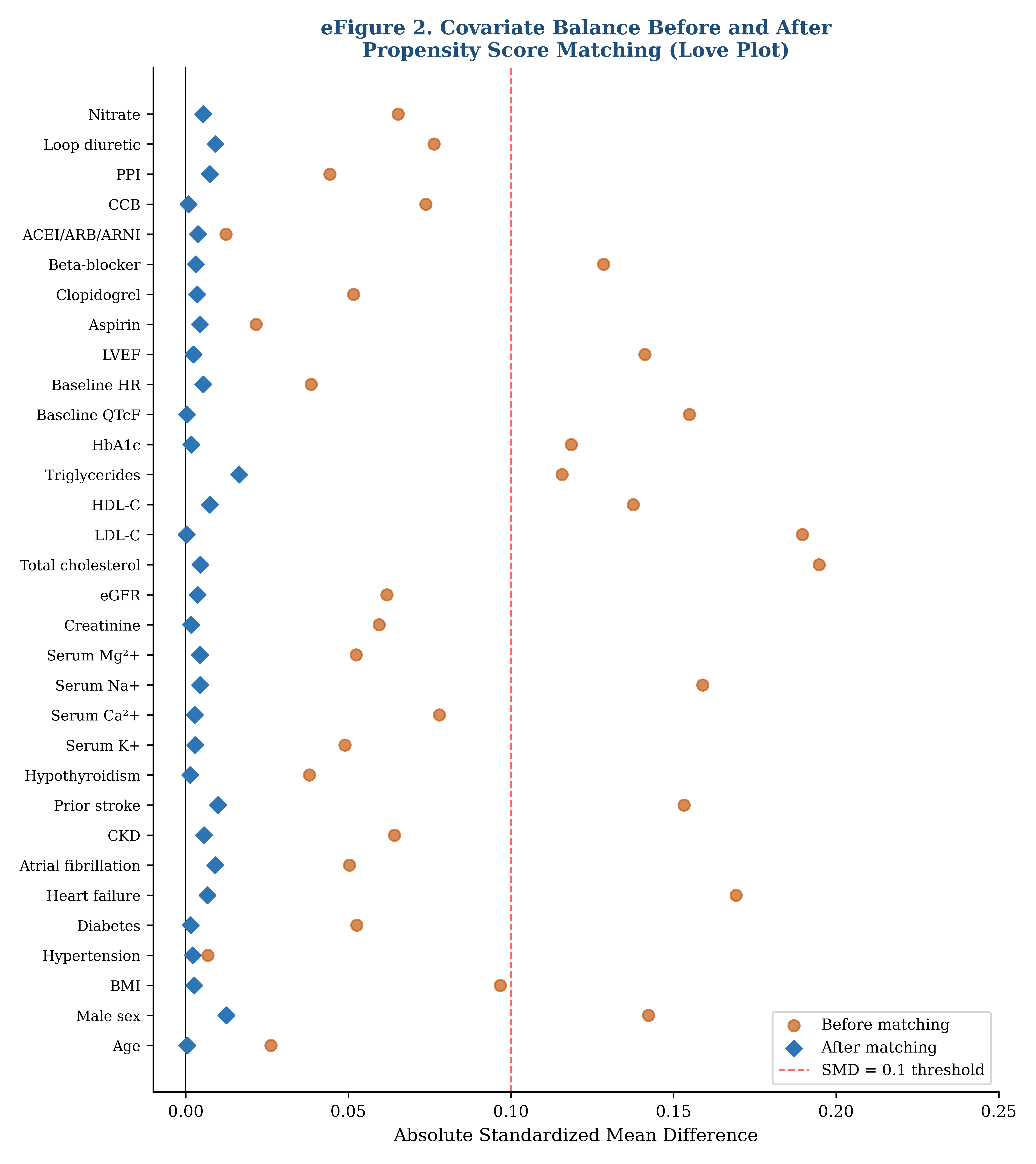
**

The plot displays absolute standardized mean differences (SMDs) for 32 baseline covariates. Orange circles represent SMDs before matching; blue diamonds represent SMDs after matching. The red dashed vertical line indicates the conventional threshold of 0.1 for adequate balance. After matching, all covariates achieved SMDs below 0.013, indicating excellent covariate balance.

**Supplementary Figure 3. Distribution of Change in QTcF Interval by Treatment Group.**

**
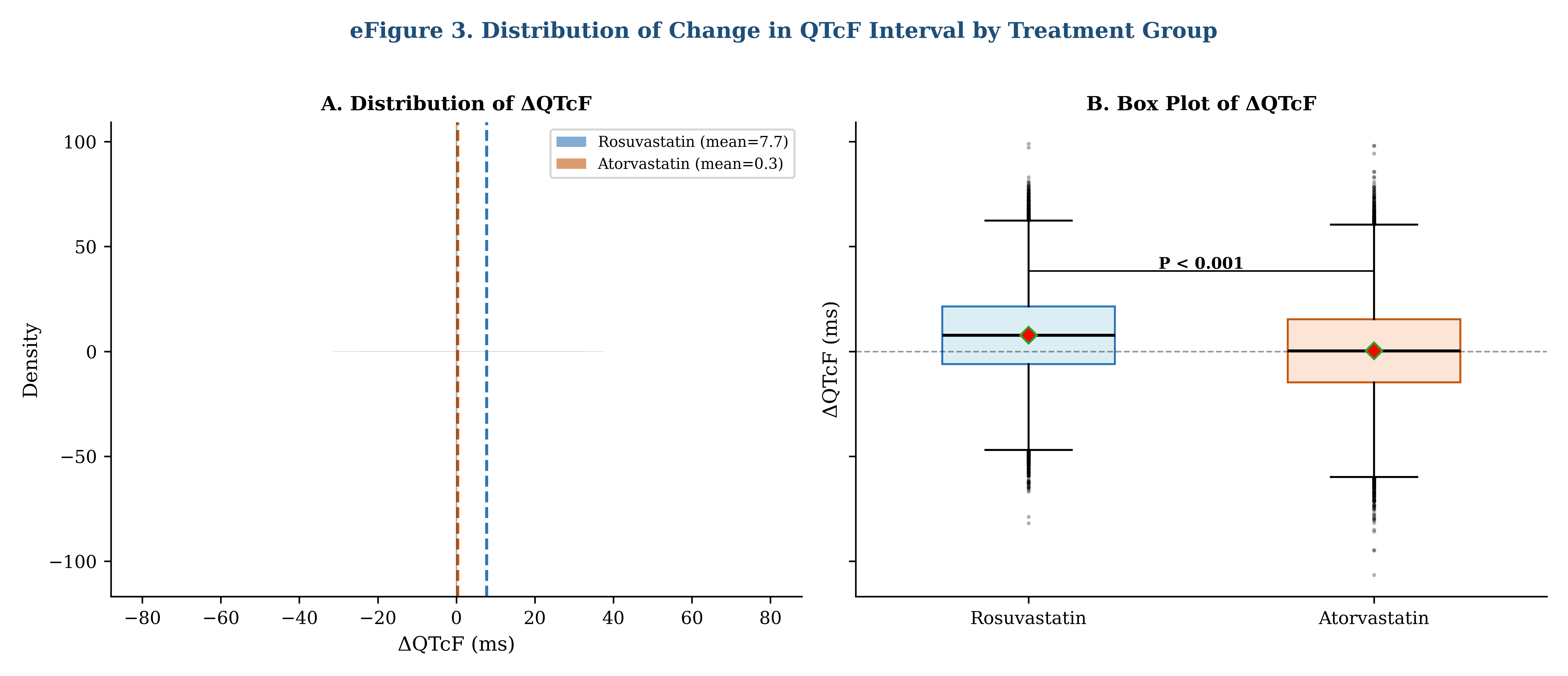
**

Panel A shows overlapping histograms of the change in Fridericia-corrected QT interval (ΔQTcF, ms) from baseline to follow-up for the rosuvastatin group (blue) and atorvastatin group (orange), with dashed vertical lines indicating group means. Panel B presents box plots with diamond markers indicating means and horizontal lines indicating medians. The rosuvastatin group shows a rightward shift (positive ΔQTcF), indicating greater QTc prolongation. P < 0.001 by independent-samples t-test.

**Supplementary Figure 4. Event Rates for Secondary Outcomes by Treatment Group (ITT).**

**
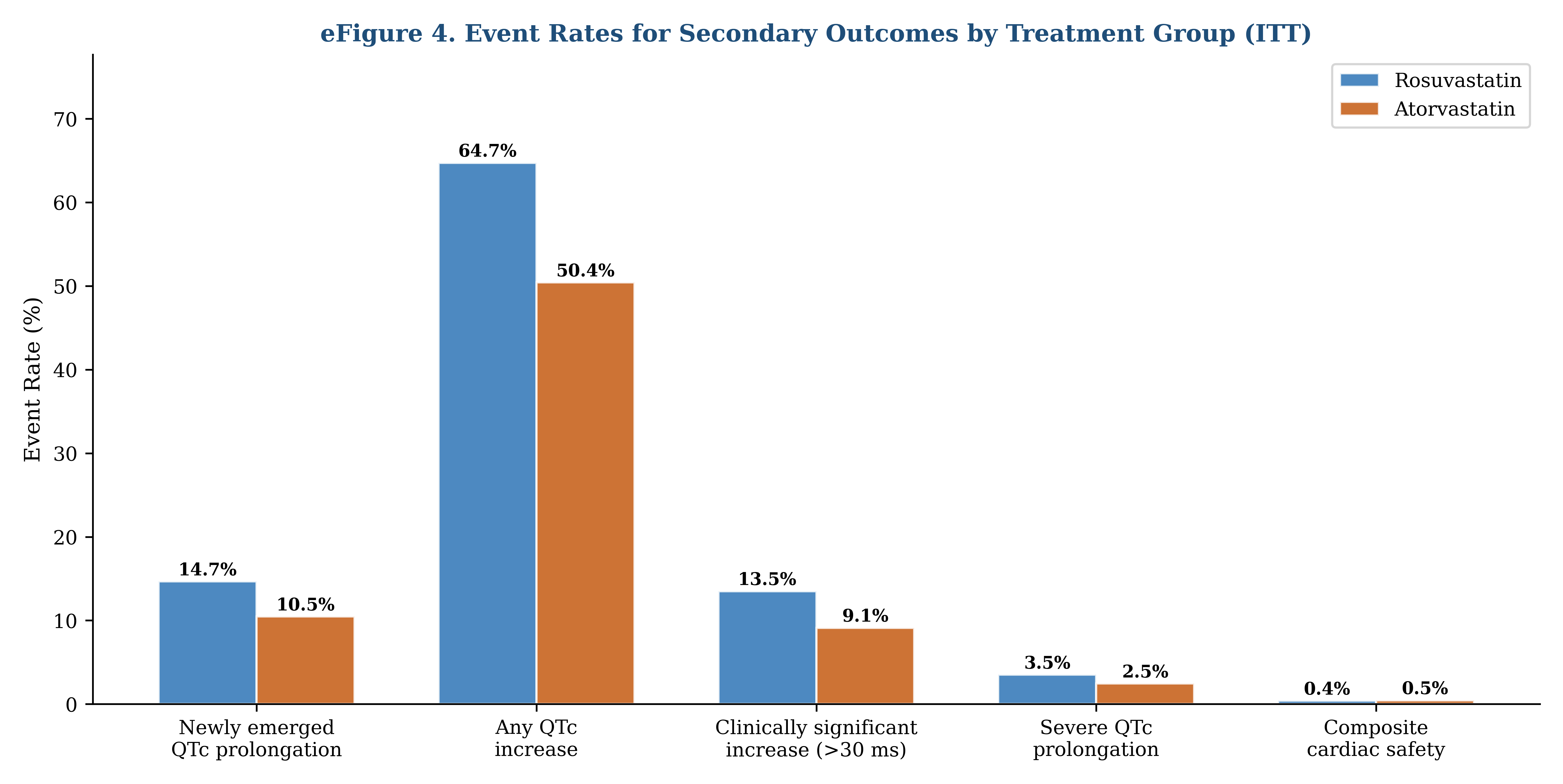
**Grouped bar chart displaying the proportion of patients experiencing each secondary outcome in the rosuvastatin group (blue) versus the atorvastatin group (orange). Percentage labels are shown above each bar. All QTc-related endpoints (newly emerged QTc prolongation, any QTc increase, clinically significant increase exceeding 30 ms, and severe QTc prolongation) were significantly more frequent in the rosuvastatin group. The composite cardiac safety endpoint did not differ significantly between groups.

**Supplementary Figure 5. Comparison of Treatment Effect Estimates: Target Trial Emulation vs Published RCT.**

**
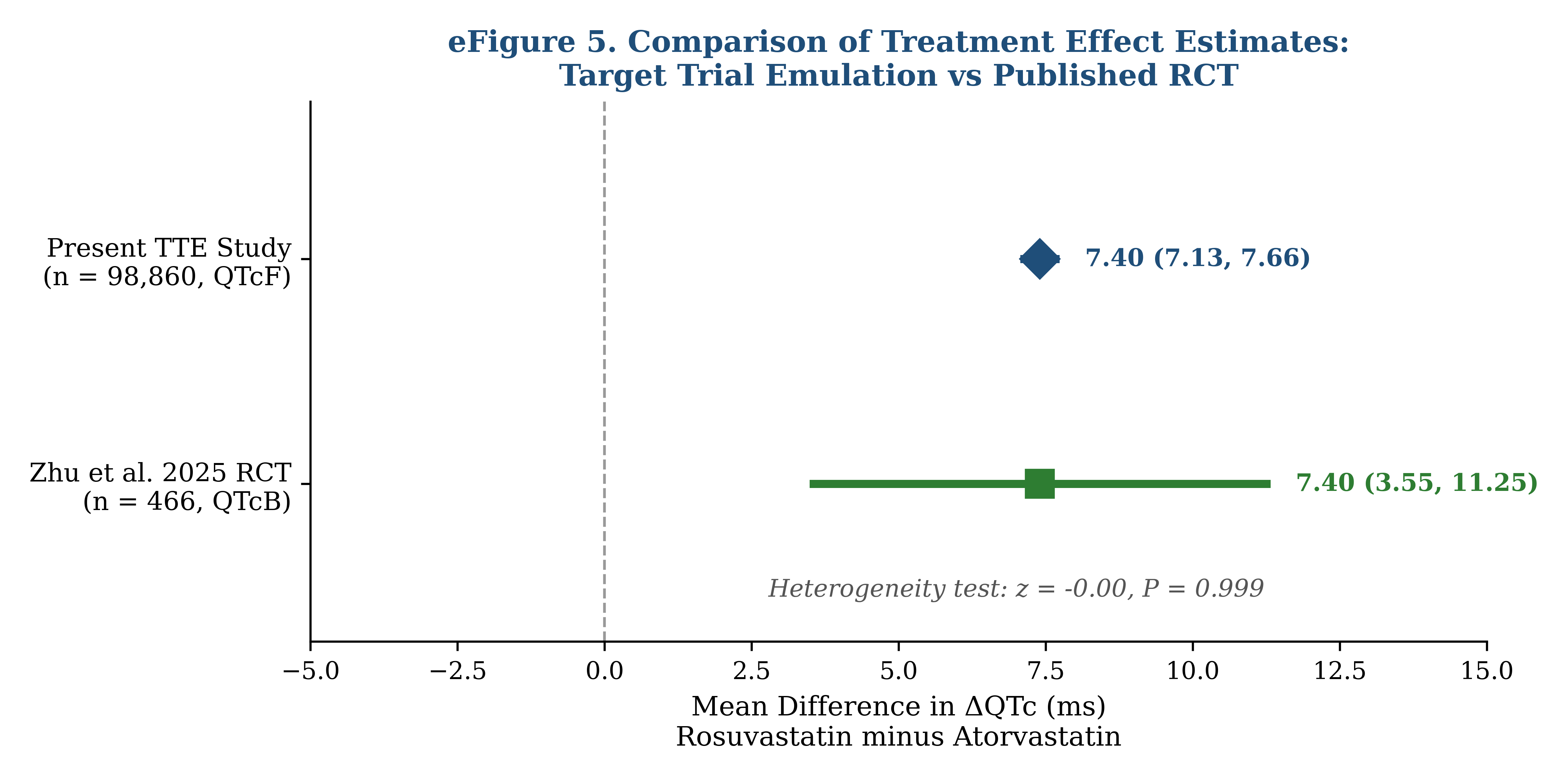
**Forest plot comparing the mean difference in QTc change (rosuvastatin minus atorvastatin, ms) between the present target trial emulation study (blue diamond; n = 98,860; QTcF, Fridericia correction) and the published randomised controlled trial by Zhu et al. 2025 (green square; n = 466; QTcB, Bazett correction). Point estimates with 95% confidence intervals are displayed. The substantially narrower confidence interval in the TTE study reflects the larger sample size. A formal heterogeneity test showed no significant difference between the two estimates (z = −0.00, P = 1.00), supporting the internal validity of the emulation approach.

# References

1. Hernán MA, Sauer BC, Hernández-Díaz S, Platt R, Shrier I. Specifying a target trial prevents immortal time bias and other self-inflicted injuries in observational analyses. J Clin Epidemiol. 2016;79:70-75.
2. Fu EL. Target trial emulation to improve causal inference from observational data: what, why, and how? J Am Soc Nephrol. 2023;34(8):1305-1314.
3. Lipsitch M, Tchetgen Tchetgen E, Cohen T. Negative controls: a tool for detecting confounding and bias in observational studies. Epidemiology. 2010;21(3):383-388.
4. VanderWeele TJ, Ding P. Sensitivity analysis in observational research: introducing the E-value. Ann Intern Med. 2017;167(4):268-274.
5. Desai RJ, Wang SV, Sreedhara SK, et al. Process guide for inferential studies using healthcare data from routine clinical practice to evaluate causal effects of drugs (PRINCIPLED). BMJ. 2024;384:e076460.
6. Li JJ, Zhao SP, Zhao D, et al. 2023 Chinese guideline for lipid management. Front Pharmacol. 2023;14:1190934.
7. Levey AS, Stevens LA, Schmid CH, et al. A new equation to estimate glomerular filtration rate. Ann Intern Med. 2009;150(9):604-612.
8. Zhu L, Shen W, Hu W, Guan H, Wang Y, Wang C, Lin J, Chen L, Jiang Q. A randomized controlled trial of the short-term effect of rosuvastatin on the corrected QT interval. Sci Rep. 2025;15(1):32076.
9. Rautaharju PM, Surawicz B, Gettes LS, Bailey JJ, Childers R, Deal BJ, et al. AHA/ACCF/HRS recommendations for the standardization and interpretation of the electrocardiogram: part IV: the ST segment, T and U waves, and the QT interval. J Am Coll Cardiol. 2009;53(11):982-991.
10. Musat DL, Adhaduk M, Preminger MW, Arshad A, Sichrovsky T, Steinberg JS, et al. Correlation of QT interval correction methods during atrial fibrillation and sinus rhythm. Am J Cardiol. 2013;112(9):1379-1383.
11. Austin PC. Balance diagnostics for comparing the distribution of baseline covariates between treatment groups in propensity-score matched samples. Stat Med. 2009;28(25):3083-3107.
12. Normand ST, Landrum MB, Guadagnoli E, Ayanian JZ, Ryan TJ, Cleary PD, et al. Validating recommendations for coronary angiography following acute myocardial infarction in the elderly: a matched analysis using propensity scores. J Clin Epidemiol. 2001;54(4):387-398.
